# Machine learning analysis of emerging risk factors for early-onset hypertension in the Tlalpan 2020 cohort

**DOI:** 10.3389/fcvm.2024.1434418

**Published:** 2025-01-17

**Authors:** Mireya Martínez-García, Guadalupe O. Gutiérrez-Esparza, Manlio F. Márquez, Luis M. Amezcua-Guerra, Enrique Hernández-Lemus

**Affiliations:** ^1^ Department of Immunology, Instituto Nacional de Cardiología Ignacio Chávez, México City, México; ^2^ Investigadora por México CONAHCYT Consejo Nacional de Humanidades, Ciencias y Tecnologías, México City, México; ^3^ Diagnostic and Treatment Division, Instituto Nacional de Cardiología Ignacio Chávez, México City, México; ^4^ Department of Electrocardiology, Instituto Nacional de Cardiología Ignacio Chávez, México City, México; ^5^ Computational Genomics Division, Instituto Nacional de Medicina Genómica, México City, México; ^6^ Center for Complexity Sciences, Universidad Nacional Autónoma de México, México City, México

**Keywords:** machine learning models, hypertension, sleep disorders, sedentary lifestyle, high-fat foods consumption, energy drink consumption, anxiety, family history

## Abstract

**Introduction:**

Hypertension is a significant public health concern. Several relevant risk factors have been identified. However, since it is a complex condition with broad variability and strong dependence on environmental and lifestyle factors, current risk factors only account for a fraction of the observed prevalence. This study aims to investigate the emerging early-onset hypertension risk factors using a data-driven approach by implementing machine learning models within a well-established cohort in Mexico City, comprising initially 2,500 healthy adults aged 18 to 50 years.

**Methods:**

Hypertensive individuals were newly diagnosed during 6,000 person-years, and normotensive individuals were those who, during the same time, remained without exceeding 140 mm Hg in systolic blood pressure and/or diastolic blood pressure of 90 mm Hg. Data on sociodemographic, lifestyle, anthropometric, clinical, and biochemical variables were collected through standardized questionnaires as well as clinical and laboratory assessments. Extreme Gradient Boosting (XGBoost), Logistic Regression (LG) and Support Vector Machines (SVM) were employed to evaluate the relationship between these factors and hypertension risk.

**Results:**

The Random Forest (RF) Importance Percent was calculated to assess the structural relevance of each variable in the model, while Shapley Additive Explanations (SHAP) analysis quantified both the average impact and direction of each feature on individual predictions. Additionally, odds ratios were calculated to express the size and direction of influence for each variable, and a sex-stratified analysis was conducted to identify any gender-specific risk factors.

**Discussion:**

This nested study provides evidence that sleep disorders, a sedentary lifestyle, consumption of high-fat foods, and energy drinks are potentially modifiable risk factors for hypertension in a Mexico City cohort of young and relatively healthy adults. These findings underscore the importance of addressing these factors in hypertension prevention and management strategies.

## 1 Introduction

Hypertension (HTN) has been well recognized as a major risk factor for morbi-mortality of cardiovascular diseases (1). Since 1990 the number of people with hypertension worldwide has doubled (2). Two decades later it has been reported that one in three people has hypertension and around 40% of adults over 25 years of age are diagnosed with hypertension worldwide (3). Hypertension has been associated to more than 10 million deaths per year, 9 million deaths from stroke, ischaemic heart disease, other vascular diseases, and renal disease, and for more than 23 million disability-adjusted life years lost worldwide (4, 5). The most recent prevalence of hypertension has been estimated around 30% to 45% among the adult population across the world and remained almost the same between men and women (6), varying by age, regions and national income (4, 7, 8).

The presence of hypertension is rising globally owing to ageing of the population and increases in exposure to lifestyle risk factors including lack of physical activity, anxiety and sleep quality (9, 10). Common determinants such as nutritional, environmental and behavioural factors of hypertension vs. normotensive at the individual level are well-established (11–13). Substance abuse such as excessive caffeine consumption through the intake of energy drinks related to other factors such as stress, excessive workload and insomnia are also proposed as emerging factors related to early-onset hypertension (14–16). On the other hand, declining mobility (17), as well as, occupational or domestic physical activity (12, 18) and transportation physical activity (19), have also been explored as factors related to develop hypertension in relatively-young adult people.

In México, the prevalence of hypertension in adults older than 20 years of age was reported to be 29.4% (27.7% in women and 31.3% in men) according to the 2022 National Health and Nutrition Survey (Ensanut 2022) (20). Of the total Mexican population with hypertension, around 47% are unaware of their diagnosis (21). However, even in the most populous city nationwide, with just over nine million inhabitants, there is still a lack of specific information regarding common and emergent hypertension risk factors, especially in relatively young adults and without any other apparent disease.

In response to the escalating prevalence of hypertension, this study has taken a data-driven approach by implementing machine learning models, including the extreme gradient boosting (XGBoost), Support Vector Machines (SVM), and Shapley Additive exPlanations (SHAP), to explore the factors associated with new-onset hypertension in initially healthy adults participating in the Tlalpan 2020 cohort study. Recent studies underscore the potential of machine learning in revealing intricate relationships within health data, particularly for populations at heightened risk of undiagnosed hypertension (22).

The implementation of models such as XGBoost in studies on hypertension factors has been a growing trend due to the algorithm’s capability to extract meaningful features and patterns. In their study, Chang et al. (23) utilized the GSFTS-FS method for feature selection and XGBoost to predict outcomes in the context of hypertension. The proposed model outperformed others by 10%, achieving high accuracy (0.95) and AUC (0.96) using cross-validation method. Additionally, Peng et al. (24) developed a hybrid model for hypertension detection, combining LASSO regression for feature selection and XGBoost for prediction. Achieved 77.2% of accuracy and 84.6% of AUC.

Moreover, techniques like SHAP have proven pivotal in hypertension prediction within machine learning (25). Miranda et al. (26) utilized SHAP to identify specific contributions of features, enhancing the performance of the Random Forest model. This improvement enabled the model to distinguish between hypertensive and normotensive patients with an accuracy of 84.2%, specificity of 78.0%, and sensitivity of 84.0%. In another study (27), researchers tackled the heightened risk of COVID-19 mortality among hypertensive patients. They employed a feature filtering algorithm for selecting relevant features and utilized SVM to predict food-derived antihypertensive peptides. The SVM model demonstrated accuracies of 86.17% and 85.61%, suggesting a promising approach for the management of hypertension.

The primary goal of this study is to explore emerging risk factors for early-onset hypertension using a data-driven approach and machine learning models within a well-established cohort in México City. Recognizing hypertension as a significant public health concern with complex and variable determinants, the research aims to identify factors beyond those currently recognized, which account for only a fraction of the observed prevalence. By analyzing data from initially healthy adults aged 18 to 50 years over a five-year follow-up period, this study employs machine learning models such as Extreme Gradient Boosting, Support Vector Machines, and Random Forest alongside Shapley values to assess the influence, direction, and contribution of a range of lifestyle, anthropometric, clinical, and biochemical variables to extend the current knowledge of hypertension’s determinants, bothbin the general case as well as in a sex-stratified analysis.

## 2 Methods

### 2.1 Study design and participants

The Tlalpan 2020 cohort is an observational, longitudinal, prospective study that was conducted at the *Instituto Nacional de Cardiología Ignacio Chávez* (INCICH), one of the National Institutes of Health and a public flagship hospital institution for the treatment of cardiovascular diseases in México. The main objective of the Tlalpan 2020 study is to evaluate the effect of traditional and non-traditional risk factors on the incidence of HTN in a cohort of México City (28).

The recruitment period was from September 2014 to June 2019. The inclusion criteria were: men and women (not pregnant or lactating) between twenty and fifty years old who live in México City; without history of cardiovascular diseases, not diagnosed with cancer with an effect on survival or with cognitive and mental disabilities; without chronic infections, inflammatory and/or immune disorders; and who agree to participate in the study. The exclusion criteria included participants identified with hypertension or diabetes during the baseline survey, as well as those who failed to provide complete information. The elimination criteria during follow up were: people who did not wish to continue participating or it was not possible to re-contact; those who changed their address outside of México City; as well as those who have developed cardiovascular disease or died.

### 2.2 Institutional review board statement

The Tlalpan 2020 study was approved by the Institutional Bioethics Committee of INCICH under number 13-802. This study was conducted according to the guidelines laid down in the Declaration of Helsinki (29).

### 2.3 Data elements and measurement scales

A set of standardized questionnaires was applied, on a face-to-face interview, to collect information on demographic characteristics: Marital status (single, married and other), highest educational level concluded (elementary school, junior high school, college and postgraduate), occupational class (student, business executive, housekeeper, professional, manually qualifies, manually unqualified, other and unemployed). Also, data of lifestyle habits and family medical history were recorded. Macro and micronutrients intake were calculated using the *Evaluation of Nutritional Habits and Nutrient Consumption System* obtained by a semi-quantitative food frequency questionnaire (SFFQ) with 140-item about dietary sources of energy, protein, carbohydrate, dietary fiber, total fat, saturated fatty acids (SFAs), monounsaturated fatty acids (MUFAs) and polyunsaturated fatty acids (PUFAs) (30). Physical activity was measured by the long version of *International Physical Activity Questionnaire, IPAQ:* categorized into low, moderate, or high physical activity levels. Psychological stress level was determined by the *State-Trait Anxiety Inventory, STAI* Spanish version (31), and sleep disorders by means of the Spanish-language Medical Outcomes Study-Sleep scale (MOS) of twelve items (32).

Anthropometric measurements (weight, height and waist circumference) were recorded during physical examination. Body weight and height were measured using a calibrated stadiometer SECA 220 and a mechanical column scale (SECA 700) with a capacity of 220 kg and precision of 0.05 kg with participants wearing light clothing and no shoes. Body mass index (BMI) was calculated as usual by taking the participant’s weight in kilograms divided by height in meters squared. Waist circumference (Waist-size) was measured at the level of 1 cm above the umbilicus. Smoking status was defined as: (1) Currently smoking, ever having smoked at least 100 cigarettes in a lifetime (Hundred-cigarettes), (2) Formerly smoked, previously smoked, had a lifetime consumption of over 100 cigarettes or currently abstains from smoking and (3) Passive smoker. Alcohol consumption was defined as the consumption of any type of alcoholic beverage at least 12 times in the last twelve months (28).

Laboratory measurements were also obtained and the biochemical data were validated in automatic analyzers at the Central Laboratory of INCICH using standardized procedures. Blood samples were obtained after an overnight fast of twelve hours (12 h). Samples were measured by Automated Photometry, Spectrophotometry, Potentiometry and Chemiluminescence and were run on the AU 680 Beckman Coulter (2012). Coulter LH Series Pak Reagent Kit. The values references of the laboratory parameters were: fasting plasma glucose (70–105 mg/dl), triglycerides (40–200 mg/dl), low-density lipoprotein cholesterol (LDL-cholesterol) (80–130 mg/dl), high-density lipoprotein cholesterol (HDL-cholesterol) (women: >50 mg/dl and men: >40 mg/dl), total cholesterol (140–200 mg/dl), uric acid (women: 3.80–6.20 mg/dl and men: 4.80–8.00 mg/dl), serum creatinine (women: 0.60–1.00 mg/dl and men: 0.70–1.30 mg/dl), Atherogenic index (LDL-cholesterol/HDL-cholesterol, elevated defined as a value >4) and serum sodium (136.00–145.00 mmol/l). Also, complete blood count parameters (counts of white blood cells, red blood cells and platelets, the mean corpuscular hemoglobin (MCH), the hematocrit and the mean platelet volume (MPV)) were examined (28)

A urine sample from a twenty-four hour (24 h) period was also obtained. For a proper urine collection, the participant was given precise and clear indications (discard the first urine in the morning and collect all urine for a period of 24 h, including the first urine of the following morning, which will be the day of the appointment). Urinary sodium and potassium were determined by the ion selective electrode method, and urinary creatinine was determined by Jaffe’s colorimetric assay using an automated analyser. The urine sample will be considered to be complete when urinary creatinine levels are within the standard creatinine excretion rate. The reference values of urinary variables are following: for creatinine in women between 740–1570 mg/24 h and for men between 1040–2350 mg/24 h, for sodium between 40.00–220.00 mmol/24 h and for potassium excretion between 25.00–125.00 mmol/24 h. Sodium and potassium excretion was reported in mmol/24 h (or equivalently mEq/24 h) (28).

### 2.4 Hypertension definition

The primary outcome of the Tlalpan 2020 study was the development of elevated blood pressure (BP) during 6,000 person-years of follow-up. The development of hypertension was defined as a previously normotensive participant whose systolic blood pressure (SBP) was ≥140 mm Hg and/or their diastolic blood pressure (DBP) was ≥90 mm Hg after at least three repeated examinations (28, 33).

### 2.5 Office blood pressure assessment

Participants were advised to have an empty bladder, not to exercise and not to smoke, drink coffee or tea, for at least 30 min before office BP measurements. Participants were seated in a quiet room (neither participant nor staff talked before, during and between measurements) with comfortable temperature for at least 5 min before the first evaluation, arm resting on table with the mid-arm at heart level; back supported on a chair; legs uncrossed and feet flat on the floor. Blood pressure was measured in the left arm three times with a three min interval between each measurement. If one of the three measurements is quite different, a four measure is taken. The value recorded is the average of the three closer measurements.

During the follow-up, all participants were contacted by telephone every 12 months to verify whether they have been diagnosed with hypertension. The main questions asked to them –based on previous studies– (34–37) were: *Has a doctor or other health provider ever told you that you have hypertension?* Participants who reported a hypertension diagnosis were then asked about current use of medication to lower BP: *Are you currently taking any medicines, tablets, or pills for hypertension?* and *In the last few weeks, have you taken any drug (medication) that could have affected your blood pressure?* In affirmative cases, all the details related to symptoms, diagnosis and pharmacological treatments are requested and documented in the medical file. We define an onset case of hypertension when those participants attend the INCICH to confirm the diagnosis by our team of clinical collaborators and to complete the “end of the study follow-up” visit format. Therefore, the date of disease onset is considered as the date of confirmation of the diagnosis or the date of anti-hypertensive treatment start. For those diagnosed with hypertension during one of the stages of follow-up visits, in every case, the date of disease onset is recorded as the date of the visit.

### 2.6 Selection of hypertensive and normotensive individuals

Of the 3,000 participants who were free of hypertension at the baseline examination, 2,500 took part in monitoring until the year 2023 at five years on average from the start of recruitment. Among them, 150 developed hypertension during follow-up and 2,350 participants remained without hypertension. For each hypertensive case, four counterparts without hypertension remained were selected throughout the same time interval and matched for gender and age (±2 years); 750 participants (150 hypertensive and 600 normotensive individuals) were eligible for final analysis.

### 2.7 Machine learning methods

**Figure 1:**
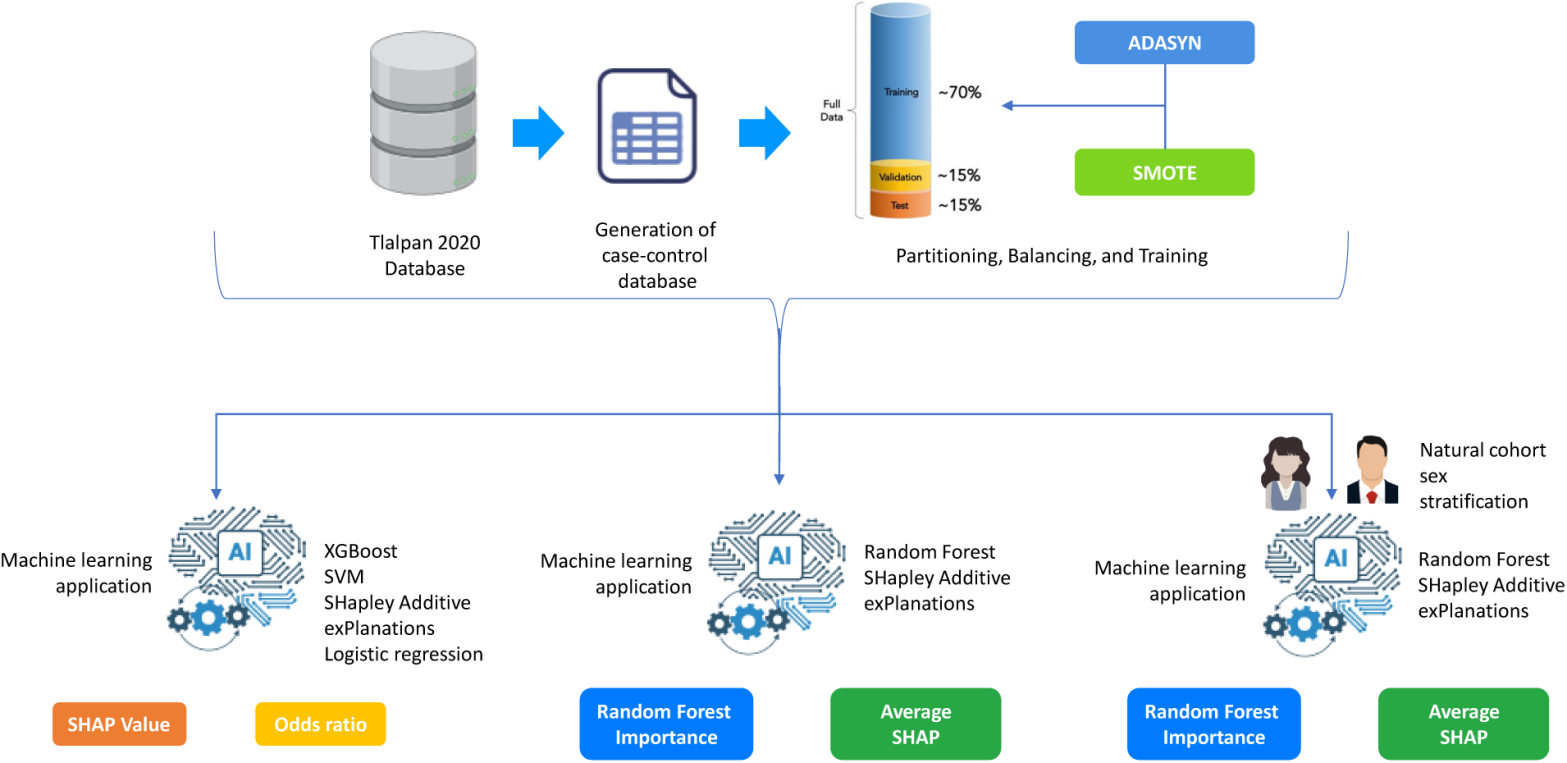
General Methodology for Hypertension Risk Prediction Using Machine Learning.

In this study, we implemented a comprehensive approach that combines various strategies to enhance the understanding of the determining risk factors related with the development of hypertension. We applied data balancing techniques and attribute selection, supported by machine learning algorithms, to identify the most relevant features (See Figure 1).

#### 2.7.1 Data balancing techniques

To counteract class distribution imbalance, mitigate associated adverse impacts, and address dimensional complexity, we implemented the techniques of SMOTE (Synthetic Minority Over-sampling Technique) and ADASYN (Adaptive Synthetic Sampling). SMOTE generates synthetic instances of the minority class by interpolating between existing samples, providing additional variability, and strengthening the representation of the less frequent class (38). Conversely, ADASYN further advances the concept introduced by SMOTE by adjusting the density of synthetic samples in proportion to the local distribution of the minority class (39). Both techniques have been widely employed to balance datasets and enhance the performance of machine learning models applied in the health sector (40, 41).

#### 2.7.2 XGBoost

After achieving a balance in the data through data balancing techniques, we applied the XGBoost algorithm not only as a machine learning model but also as a feature selection tool. XGBoost belongs to the ensemble model category, utilizing decision trees as base components. Mathematically, XGBoost’s training involves optimizing a cost function, quantifying the discrepancy between model predictions and actual values (42).

#### 2.7.3 Support vector machines (SVM)

Support Vector Machine (43) operates by finding the optimal hyperplane that maximizes the separation between classes in a multidimensional space. It transforms the data into a higher-dimensional feature space, seeking a hyperplane that maximizes the margin between classes. In this study, SVM was utilized with a radial kernel. The choice of the radial kernel was based on observed results, demonstrating improved performance according to the balanced accuracy metric.

#### 2.7.4 SHapley Additive exPlanations (SHAP)

SHapley Additive exPlanations is a concept from game theory (44) that has been successfully applied to assess the contribution of each feature to a model’s predictions. In this study, Shapley Values were employed to identify features related to hypertension. The subset of features obtained through Shapley Values was subsequently evaluated using a support vector machine with a radial kernel (see Equation 1). The Shapley Values are defined by:

(1) $$\phi_{i}(v)=\sum_{S\subseteq N\setminus \{i\}}\frac{|S|!(|N|-|S|-1)!}{|N|!}\left(v(S\cup \{i\})-v(S)\right)$$

Where:$\phi_{i}(v)$ is the Shapley Value of player. N is the set of players. v is the characteristic function assigning a value to each coalition of players. S is a coalition of players that does not contain player i. |S| is the number of players in coalition S. |N| is the total number of players.

#### 2.7.5 Random forest

Random Forest, developed by Leo Breiman in 2001, is a machine learning algorithm that combines multiple decision trees to improve accuracy and reduce overfitting. By creating a forest of decision trees, each trained on a random subset of the data and features, the model makes a final prediction based on majority voting. In Python, key parameters such as *n_estimators* (the number of trees), *max_depth* (the maximum depth of each tree), *min_samples_split* (the minimum number of samples required to split a node), and *max_features* (the maximum number of features considered at each split) allow fine-tuning of the model to optimize performance and reduce overfitting. A key feature of Random Forest is its ability to calculate Variable Importance Percent, which quantifies each feature’s contribution by measuring the reduction in impurity across all trees. Higher importance scores indicate a stronger influence on predictions, highlighting the most impactful variables.

#### 2.7.6 Performance evaluation metrics

Sensitivity (SEMS) measures the model’s ability to correctly identify positive instances, while specificity (SPC) assesses its ability to accurately classify negative instances. Balanced accuracy (B.ACC) considers both proportions, providing a comprehensive measure of the model’s overall performance. In health contexts, where the accurate identification of both positive and negative cases is critical, these metrics provide a comprehensive view of the model’s performance (see Equations 2–4). The equations are defined by: ?>

(2) $$\text{SEMS}=\frac{\text{TP}}{\text{TP}+\text{FN}}$$

(3) $$\text{SPC}=\frac{\text{TN}}{\text{FP}+\text{TN}}$$

(4) $$\text{B.ACC}=\left(\frac{1}{2}\right)\left(\frac{\text{TP}}{\text{P}}+\frac{\text{TN}}{\text{N}}\right)$$

In this context, P represents positive instances, N represents negative instances, and TP, FN, TN, and FP denote true positives, false negatives, true negatives, and false positives, respectively.

The Jupyter Notebook environment (version 6.4.12) was employed for developing and evaluating machine learning models, as well as designing network calculators, using Python (version 3.9.13). For handling class imbalance in the dataset, both ADASYN and SMOTE were implemented. Additionally, SHAP was utilized for feature importance and selection. The computer equipment utilized consisted of a Dell workstation, featuring an Intel(R) Xeon(R) Core processor, 32 GB of RAM, and a processor speed of 3.50 GHz, with Windows operating as the system.

### 2.8 Statistical analysis

The baseline characteristics of hypertensive and normotensive individuals were compared using Pearson’s chi-squared test for categorical variables and Wilcoxon test for continuous variables. The distribution of numerical data was assessed using the Shapiro-Francia tests. Analyses were performed using [R] version 4.0.2 (45).

#### 2.8.1 Experimental design configuration

Firstly, data balancing techniques were implemented with the aim of ensuring optimal performance of the models and feature selection. This phase addressed potential imbalances in the class distribution, ensuring that the models could learn equitably from all instances and, consequently, enhance their overall performance. Subsequently, a dataset partition was conducted, allocating 70% for training and 30% for testing. Additionally, a 10-fold cross-validation strategy was configured. This choice is grounded in the need to obtain more reliable estimations of the model’s performance by training and evaluating it repeatedly on different subsets of data.

For parameter selection after configuring the 10-fold cross-validation, a grid method was employed. For XGBoost, the following parameter grid in was varied: max_depth, learning_rate, and n_estimators. Similarly, for SVM with kernel RBF, the parameter grid included C and gamma. This additional step aims to find the optimal parameter combination that maximizes the predictive performance of the models. Finally, the selected subsets of features and parameters were put to the test in the models. A similar experimental design was implemented for the sex-stratified analysis.

## 3 Results

### 3.1 General characteristics of the participants

The primary outcome was the development of hypertension during 6,000 person-years of follow-up. Of the 150 participants who developed hypertension 61.33% was women of median age 50 (47–55 IQR) and 38.67% was men of median age 48 (42–53 IQR) (see Supplementary Table S1). Six hundred normotensive participants were selected to match them by sex and age. Once the data of the sociodemographic, anthropometric, family pathological history, lifestyles and clinical evaluations were obtained and compared –between men and women with and without hypertension– some features stand out: The majority of women with hypertension were classified in the low SDI group (24%), while the highest proportion of men with hypertension was found in the high SDI group (18%). Despite these differences, both groups had a higher prevalence of individuals with advanced education and employment in skilled or professional occupations (see Supplementary Table S2).

A higher percentage of smokers (active or passive) was reported in cases of hypertension, 32% vs. 29.88% in smoking women and 32% vs. 29.38% in smoking men (see Supplementary Table S1). Most of the participants –hypertensive and normotensive –apparently in good health were overweight, BMI of women was 28.46 (25.97–34.22 IQR) vs. 26.59 (23.80–29.72 IQR), and BMI of men was 28.46 (26.83–31.16 IQR) vs. 26.91 (24.21–29.92 IQR), see Supplementary Table S1.

Women and men with new-onset hypertension reported a higher percentage of risk factors than normotension such as: father with obesity, 12% and 8.67%; smoking mother, 6.67% and 8%; mother with diabetes, 18.67% and 8.67%; father with diabetes, 21.33% and 8%; mother with hypertension, 28% and 14.67%; father with hypertension, 19.33% and 12.67% –see Supplementary Table S3. A greater number of minutes spent sitting at day was reported among cases that developed hypertension vs. those who remained normotensive: 300 min (180–420 IQR) vs. 240 min (120–360 IQR) in women and 300 min (180–480 IQR) vs. 240 min (180–480 IQR) in men (see Supplementary Table S4).

Clinical parameters with a statistically significant difference between hypertensive and normotensive were: HDL-cholesterol mg/dl 47 vs. 49.35 ($p_{\text{value}}=0.0359$) in women and 38.85 vs. 41.50 ($p_{\text{value}}=0.0118$) in men; and the triglycerides only between men 194.85 vs. 153.90 ($p_{\text{value}}=0.0077$) (see Supplementary Table S4). The Supplementary Materials provide additional descriptive data on the cohort, including details on sociodemographic factors, anthropometric measurements, family history of disease, sleep characteristics, physical activity, psychological stress, and the consumption of tobacco, alcohol, or energy drinks. They also include information on basic clinical parameters, food and nutrient consumption reports and complete blood count results.

### 3.2 Machine learning results

To deepen the evaluation of the model’s performance in predicting hypertension, three analyses were conducted to assess the influence, direction, and relative importance of individual features that improve the model’s predictive accuracy from the natural cohort (prior to matching). Subsequently, a sex-stratified analysis was performed to identify potential gender-specific risk factors. To illustrate the methodological workflow implemented in this study, Figure 1 provides an overview of the steps taken to analyze hypertension risk factors. The process begins with the Tlalpan 2020 Database, a comprehensive cohort dataset. A case-control dataset was generated to identify individuals with hypertension (cases) and matched controls. The data was then split into training (70%), validation (15%), and test (15%) sets. To address class imbalance, ADASYN and SMOTE were applied, ensuring balanced data for model training and more accurate feature importance assessment. These preprocessing steps established a solid foundation for predictive modeling and the identification of key hypertension risk factors.

The first analysis identifies the most important features based on their Shapley values, applying XGBoost and SVM algorithms along with resampling techniques such as ADASYN and SMOTE to balance the dataset. This step provides an initial understanding of the variables that hold the greatest influence in predicting hypertension, as determined by their Shapley contribution scores.

In the second analysis, Random Forest was employed to further validate the key features, using its Variable Importance Percent metric to highlight the most influential variables. Additionally, Shapley values were applied once more to these Random Forest results, allowing for a more detailed examination of each feature’s direction and level of contribution to hypertension risk. This combination of Random Forest importance scores and Shapley values enables a comprehensive view of whether each variable positively or negatively impacts hypertension prediction and to what extent.

Lastly, a gender-specific analysis was conducted by applying Random Forest separately for men and women to capture potential differences in feature importance by sex. Using Shapley values again in this gender-stratified analysis, along with ADASYN and SMOTE balancing techniques, this final evaluation reveals how each variable uniquely contributes to hypertension risk for men and women, adding another layer of interpretability to the model.

#### 3.2.1 Feature importance analysis using extreme gradient boosting and support vector machines

In this section, we present the results of the most relevant variables identified using XGBoost and SVM, analyzed with SHAP values, in conjunction with ADASYN and SMOTE balancing techniques. The results include each variable’s average SHAP value, odds ratio, direction of influence (positive or negative), and an interpretation of the impact on hypertension risk.

**Figure 2:**
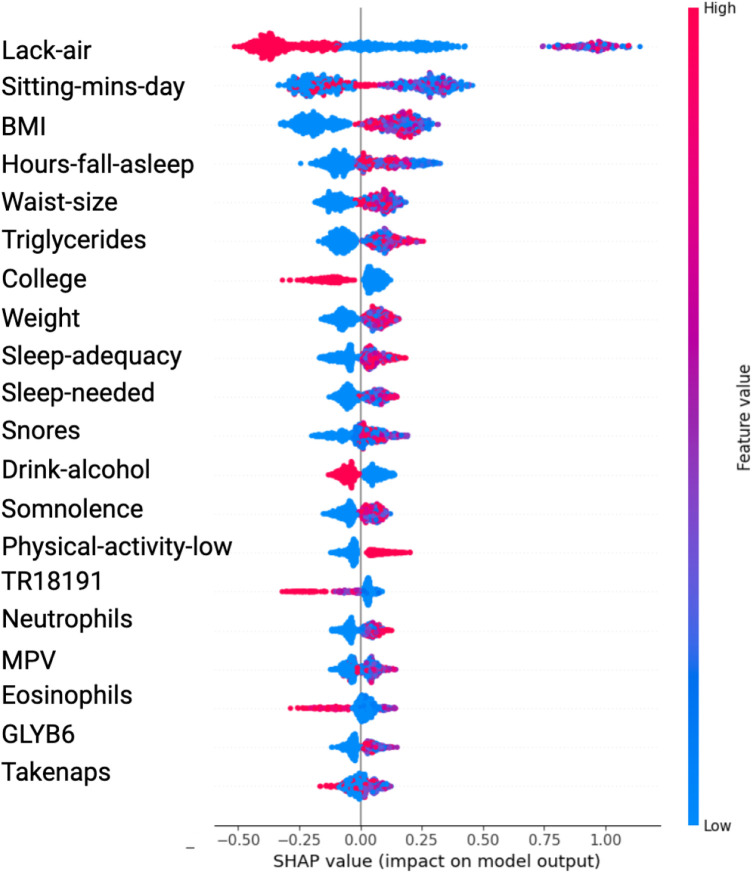
Variable Significance with XGBoost, SHAP and ADASYN. *Lack-air*, Waking up feeling short of breath and/or headache; *Sitting-mins-day*, Minutes sitting per day; *Waist-size*, Waist circumference; *BMI*, Body mass index; *MPV*, Mean platelet volume; *Sleep-adequacy*, Adequate sleep report; *Trait-anxiety*, Anxiety as a trait; *State-anxiety*, Anxiety as a state; *SS-Headache*, Hours-fall-asleep and wake up with a headache; *Low-physical-act*, Low physical activity; *Hours-fall-asleep*, Hours to fall asleep; *Take-naps*, Take-naps of more than five minutes a day; *SDI-Level*, Social Development Index.

**Figure 3:**
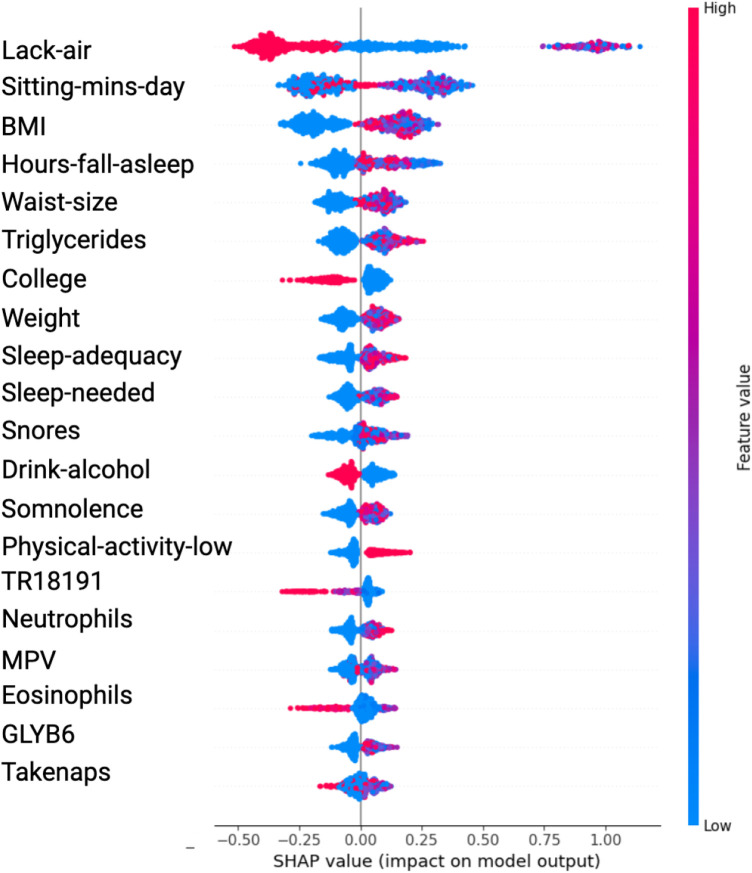
Variable Significance with XGBoost, SHAP and SMOTE *Lack-air*, Waking up feeling short of breath and/or headache; *Sitting-mins-day*, Minutes sitting per day; *BMI*, Body mass index; *Hours-fall-asleep*, Hours to fall asleep; *Waist-size*, Waist circumference; *Sleep-adequacy*, Adequate sleep report; *Sleep-needed*, Not getting enough sleep to feel rested when wake up in the morning; *TR18191*, Trans 18:1 fatty acid; *MPV*, Mean platelet volume; *Take-naps*, Take-naps of more than five minutes a day.

**Figure 4:**
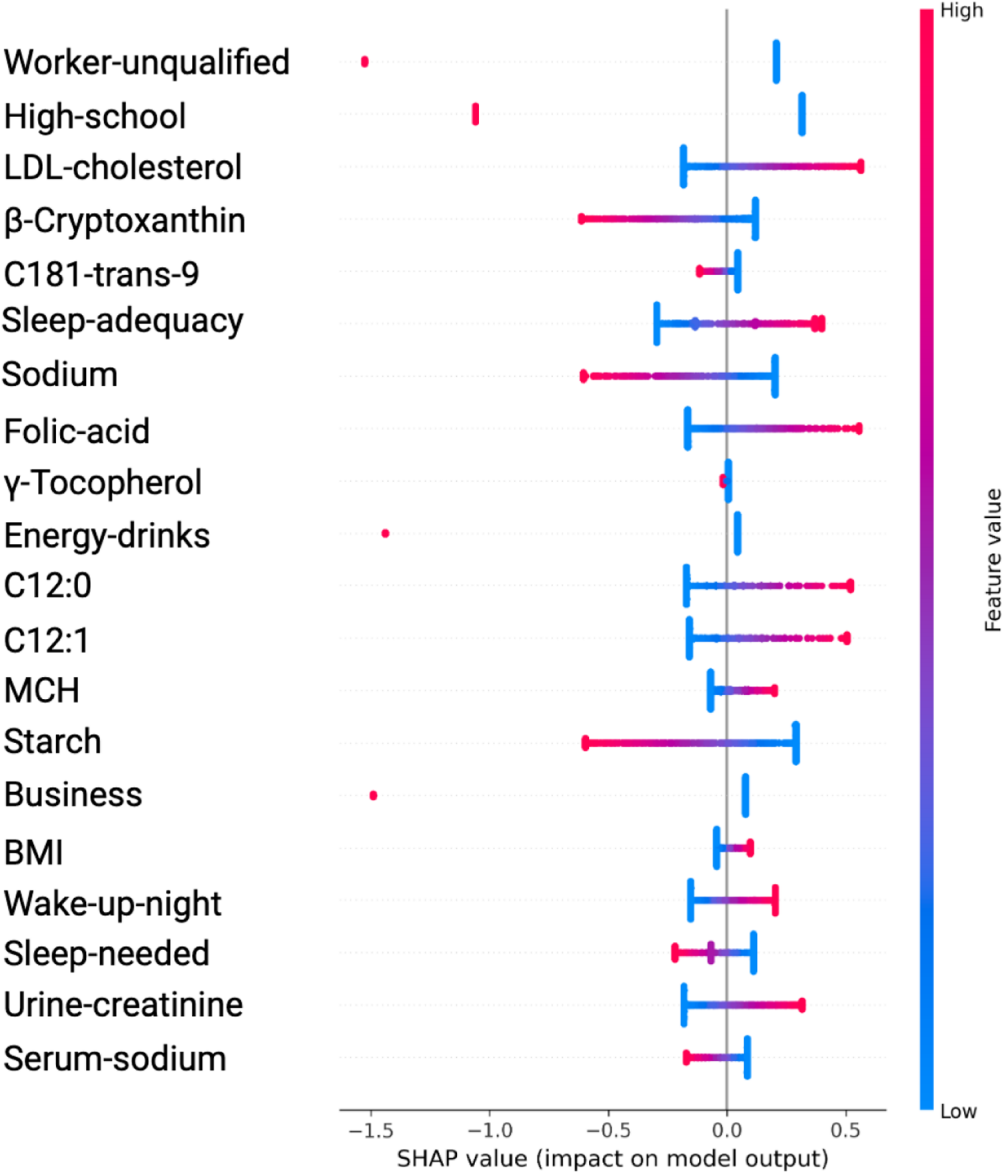
Variable Significance with SVM, SHAP and ADASYN. *C181-trans-9*, unsaturated trans fatty acid; *MCH*, mean corpuscular hemoglobin; *C12:0 and C12*, saturated fatty acids; *Sleep-adequacy*, Adequate sleep report; *Sleep-needed*, Not getting enough sleep to feel rested when wake up in the morning; β*-Cryptoxanthin*, carotenoids; γ*-Tocopherol*, tocopherols; *Wake-up-night*, Wakes up while sleeping and has difficulty going back to sleep.

**Figure 5:**
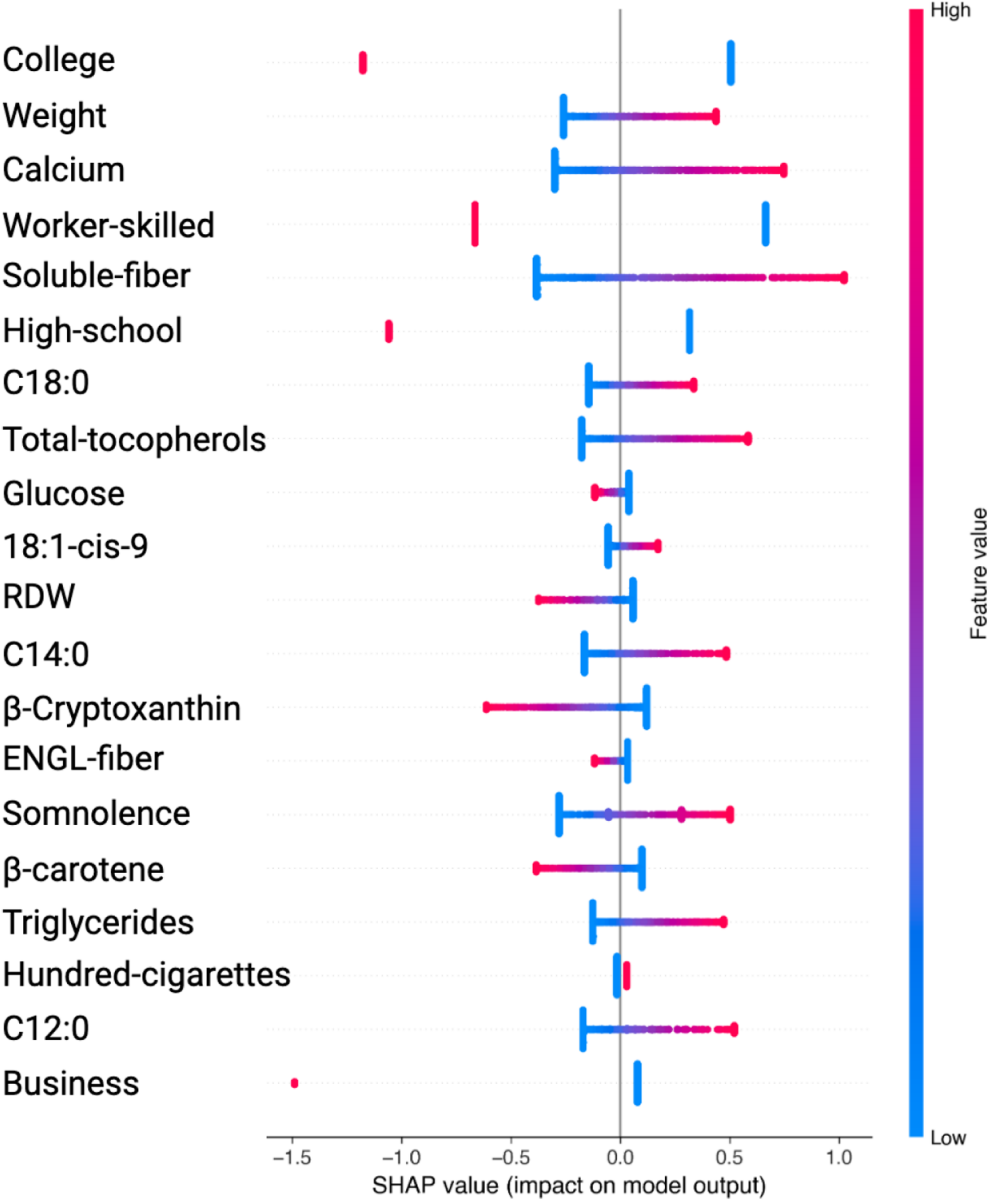
Variable Significance with SVM, SHAP and SMOTE. *C18:0, C12:0, C14:0*, specific saturated trans fatty acid; *18:1-cis-9*, monounsaturated fatty acids omega-9; *ENGL-fiber*, dietary fiber; *Hundred-cigarettes*, consumption of more than 100 cigarettes; *RDW*, Red Blood Cell Distribution Widht.

The SHAP value plots (Figures 2, 3, 4, 5) visually represent the impact of each feature on the model’s predictions, where each point corresponds to an individual feature’s contribution. SHAP values along the x-axis indicate both the direction and magnitude of this influence: points extending further to the right suggest an increase in the model’s prediction, while those on the left indicate a decrease. High feature values are depicted in red, and their distance from the center (left or right) highlights the most influential features. Figure 2 shows the SHAP values for features balanced with ADASYN using XGBoost, while Figure 3 presents the features balanced with SMOTE in XGBoost. Figure 4 highlights the features identified by SVM with ADASYN, and Figure 5 displays those obtained by SVM with SMOTE.

Table 1 presents the significant variables identified through XGBoost and ADASYN, showing the average SHAP value and the odds ratio for each feature. The average SHAP value reflects the mean impact of each feature on the model’s predictions, while the odds ratio provides a quantitative measure of both the direction and strength of its influence. According to this table, the variables such as Lack-air (waking up feeling short of breath or with a headache), BMI and Waist-size, have a high SHAP value but an odds ratio of 0.1795, 0.5708 and 0.8577 respectively, indicating a reduction in hypertension risk for individuals with this feature. In contrast, Sitting-mins-day (minutes sitting per day) and Weight as well as Low-physical-activity and Hours-fall-asleep also have a high SHAP value but an odds ratio above 1, suggesting that sedentary behavior and obesity increases the risk of hypertension. Also, the Table 1 indicate that anxiety levels, whether as a personality trait (Trait-anxiety), has a positive influence on hypertension risk, with odds ratio of 2.5114. This suggests that persistent anxiety levels may be key factor in hypertension development.

**Table 1:** Features obtained by XGBoost and SHAP using ADASYN.

Feature	Shapley_Value_Mean	Odds_Ratio	Direction	Interpretation
Lack-air	0.3098	0.1795	Negative	Decreases risk
Sitting-mins-day	0.1542	1.5408	Positive	Increases risk
Waist-size	0.1013	0.5708	Negative	Decreases risk
BMI	0.0821	0.8577	Negative	Decreases risk
Weight	0.0728	5.6916	Positive	Increases risk
MPV	0.0565	1.1925	Positive	Increases risk
Sleep-adequacy	0.0540	2.3522	Positive	Increases risk
Trait-anxiety	0.0502	2.5114	Positive	Increases risk
Serum-sodium	0.0490	1.3892	Positive	Increases risk
State-anxiety	0.0471	0.6596	Negative	Decreases risk
SS-Headache	0.0437	0.7475	Negative	Decreases risk
Physical-activity-low	0.0400	1.8930	Positive	Increases risk
Hours-fall-asleep	0.0364	1.3908	Positive	Increases risk
Vitamin-B6	0.0361	0.5001	Negative	Decreases risk
Caproic-acid	0.0356	0.5159	Negative	Decreases risk
Take-naps	0.0344	0.9254	Negative	Decreases risk
Drink-alcohol	0.0333	0.4578	Negative	Decreases risk
Mother-with-obesity	0.0316	0.2625	Negative	Decreases risk
Mother-with-dyslipidemia	0.0308	0.3568	Negative	Decreases risk
SDI-Level	0.0296	0.5930	Negative	Decreases risk

In the results obtained with SVM using SMOTE technique (see Figure 3 and Table 2), Lack-air emerges as the most significant feature, evidenced by a high SHAP value and a large cluster of red points on the right side of the plot. Despite its high impact in SHAP, the odds ratio for Lack-air is 0.1845, indicating a decreased risk of hypertension for individuals with this feature. Other sleep-related variables, such as Hours-fall-asleep, Sleep-adequacy, Snores, Somnolence and Take-naps also play prominent roles, reflecting a strong association between sleep quality and hypertension risk.

**Table 2:** Features obtained by XGBoost and SHAP using SMOTE.

Feature	Shapley_Value_Mean	Odds_Ratio	Direction	Interpretation
Lack-air	0.3582	0.1845	Negative	Decreases risk
Sitting-mins-day	0.2163	1.0579	Positive	Increases risk
BMI	0.1731	0.8083	Negative	Decreases risk
Hours-fall-asleep	0.1067	2.0393	Positive	Increases risk
Waist-size	0.0919	0.6031	Negative	Decreases risk
Triglycerides	0.0904	1.3844	Positive	Increases risk
College	0.0791	0.3325	Negative	Decreases risk
Weight	0.0728	6.1512	Positive	Increases risk
Sleep-adequacy	0.0637	1.5636	Positive	Increases risk
Sleep-needed	0.0622	0.5748	Negative	Decreases risk
Snores	0.0589	1.5819	Positive	Increases risk
Drink-alcohol	0.0574	0.4160	Negative	Decreases risk
Somnolence	0.0548	1.4142	Positive	Increases risk
Physical-activity-low	0.0533	2.1273	Positive	Increases risk
TR18191	0.0518	0.2979	Negative	Decreases risk
Neutrophils	0.0486	1.1002	Positive	Increases risk
MPV	0.0450	1.4174	Positive	Increases risk
Eosinophils	0.0450	0.5932	Negative	Decreases risk
Vitamin-B6	0.0421	0.9242	Negative	Decreases risk
Take-naps	0.0371	1.0729	Positive	Increases risk

Again in this model expected variables such as Sitting-mins-day, Low-physical-activity and Weight show a rightward spread, emphasizing the impact of sedentary behavior and obesity-related factors on hypertension, with high SHAP values and positive odds ratios indicating increased risk. Triglycerides, with a positive odds ratio of 1.3844, aligns with cardiovascular risk. Lower-ranking features such as Neutrophils, Eosinophils, and MPV also contribute positively, indicating their relevance in hypertension prediction.

In the case of features identified by SVM with ADASYN or SMOTE, they showed significant variations to compared to those in Figures 2 and 3. This discrepancy stems from the SVM models’ non-tree-based structure, prompting SHAP to adopt a permutation-based approach for calculating values, which typically yields a single SHAP value for each feature. In contrast, when using tree-based models such as XGBoost, SHAP utilizes TreeExplainer, a method specifically designed for these models. This approach facilitates the computation of detailed SHAP values for every feature, thereby offering a more nuanced depiction of how each feature impacts the model’s predictions.

The analysis using SVM and ADASYN (see Figure 4 and Table 3) identified features like Worker-unqualified, High-school, LDL-cholesterol, Cryptoxanthin, and C1S1891 as top predictors for the model of hypertension risk.

**Table 3:** Features obtained by SVM and SHAP using ADASYN.

Feature	Shapley_Value_Mean	Odds_Ratio	Direction	Interpretation
Worker-unqualified	0.0572	0.4762	Negative	Decreases risk
High-school	0.054	0.7611	Negative	Decreases risk
LDL-cholesterol	0.0454	0.5921	Negative	Decreases risk
Cryptoxanthin	0.0323	0.4998	Negative	Decreases risk
C1S1891	0.0323	0.5576	Negative	Decreases risk
Sleep-adequacy	0.0286	1.6923	Positive	Increases risk
Sodium	0.0237	0.4805	Negative	Decreases risk
Folic-acid	0.0219	0.6850	Negative	Decreases risk
Tocopherol	0.0217	1.4804	Positive	Increases risk
Energy-drinks	0.0212	0.1474	Negative	Decreases risk
C12:0	0.0209	0.5945	Negative	Decreases risk
C12:1	0.0184	1.7331	Positive	Increases risk
MCH	0.0181	0.6747	Negative	Decreases risk
Starch	0.0176	1.6879	Positive	Increases risk
Business	0.0176	0.2789	Negative	Decreases risk
BMI	0.0171	1.7755	Positive	Increases risk
Wake-up-night	0.0169	0.8225	Negative	Decreases risk
Sleep-needed	0.0164	0.8276	Negative	Decreases risk
Urine-creatinine	0.0163	1.5697	Positive	Increases risk
Serum-creatinine	0.0162	0.6927	Negative	Decreases risk
Serum-sodium	0.0156	1.4802	Positive	Increases risk

Similarly, in the SVM analysis with SMOTE, key features such as College, Weight, Calcium, Worker-skilled, Soluble-fiber, and High-school show high SHAP values, indicating a strong impact on the model’s predictions (see Figure 5 and Table 4).

**Table 4:** Features obtained by SVM and SHAP using SMOTE.

Feature	Shapley_Value_Mean	Odds_Ratio	Direction	Interpretation
College	0.1185	0.2408	Negative	Decreases risk
Weight	0.0783	3.3324	Positive	Increases risk
Calcium	0.0723	0.9265	Negative	Decreases risk
Worker-skilled	0.0556	0.4268	Negative	Decreases risk
Soluble-fiber	0.0498	1.0214	Positive	Increases risk
High-school	0.0496	0.4787	Negative	Decreases risk
C18:0	0.0436	0.6798	Negative	Decreases risk
Total-tocopherols	0.0415	1.1783	Positive	Increases risk
Glucose	0.0388	0.7673	Negative	Decreases risk
18:1-cis-9	0.0375	0.9970	Negative	Decreases risk
RDW	0.0356	1.1463	Positive	Increases risk
C14:0	0.0334	0.4052	Negative	Decreases risk
Cryptoxanthin	0.0309	0.5285	Negative	Decreases risk
ENGL-fiber	0.0309	0.9303	Negative	Decreases risk
Somnolence	0.0304	1.3639	Positive	Increases risk
Bcarotene	0.0301	0.8873	Negative	Decreases risk
Triglycerides	0.0294	1.4773	Positive	Increases risk
Hundred-cigarettes	0.0289	0.6233	Negative	Decreases risk
C12:0	0.0275	1.6386	Positive	Increases risk
Business	0.026	0.3376	Negative	Decreases risk

Notably, although these features exhibited high SHAP values, their odds ratios were below 1, prompting further analysis to better understand their behavior across different algorithms.

In the ADASYN analysis, Urine-creatinine, Serum-sodio and Sleep-adequacy were found to have odds ratios significantly above 1, suggesting that these factors are closely linked to an increased hypertension risk. Their high odds ratios imply a more tangible and direct contribution to hypertension risk compared to features identified with high SHAP values alone.

For the SMOTE analysis, variables such as Weight, Somnolence, Triglycerides and others were marked by high odds ratios, making them essential to predict hypertension risk.

In summary, the comparative analysis of XGBoost and SVM using different balancing techniques and feature selection with Shapley Values demonstrates that the XGBoost model combined with SMOTE achieved the highest balanced accuracy at 90%, with a sensitivity of 0.8448 and a specificity of 0.9623 (see Table 5). This result indicates that the XGBoost-SMOTE configuration provides the best overall performance for hypertension prediction in this study, balancing the ability to correctly identify both positive and negative cases. Other models, including XGBoost with ADASYN and SVM with both ADASYN and SMOTE, also showed respectable performance but did not reach the same level of balanced accuracy.

**Table 5:** Comparative performance of Machine Learning Models.

Model	Parameters	Feature selection technique	Balancing technique	Sensibility	Specificity	Balanced Acuracy
	learning_rate = 0.1,					
XGBoost	max_depth = 7,	Shapley Value	SMOTE	0.8448	0.9623	90%
	n_estimators = 300					
	learning_rate = 0.01,					
XGBoost	max_depth = 9,	Shapley Value	ADASYN	0.8135	0.9677	89%
	n_estimators = 200					
SVM - RBF	C=10, gamma = 2	Shapley Value	SMOTE	0.7758	0.8709	82%
SVM - RBF	C=100, gamma = 2	Shapley Value	ADASYN	0.8249	0.9086	87%
	ntree = 200,					
RF	max_depth = 20	Shapley Value	ADASYN	0.8647	0.9846	92%
	max_features = sqrt	Importance Percent				
	ntree = 200,					
RF	max_depth = 20,	Shapley Value	SMOTE	0.8248	0.9945	90%
	max_features = log2	Importance Percent				
	ntree = 200,					
RF (women)	max_depth = 10,	Shapley Value	ADASYN	0.7777	0.9561	86%
	max_features = sqrt	Importance Percent				
	ntree = 200,					
RF (women)	max_depth = 10,	Shapley Value	SMOTE	0.8207	0.9826	90%
	max_features = log2	Importance Percent				
	ntree = 100,					
RF (men)	max_depth = None,	Shapley Value	ADASYN	0.8024	0.9344	86%
	max_features = sqrt	Importance Percent				
	ntree = 100,					
RF (men)	max_depth = None,	Shapley Value	SMOTE	0.8414	0.9814	92%
	max_features = log2	Importance Percent				

Given the results obtained, a further analysis was conducted using the Random Forest algorithm. Unlike the previous analyses, which utilized odds ratios to establish variable contributions, this approach applied the variable importance metric within Random Forest to identify the most significant features. To complement this, Average SHAP values were employed to determine the direction and contribution of each variable whether they increase or decrease hypertension risk. The choice to use SHAP values instead of odds ratios in this phase was intentional, as SHAP values are specifically designed to explain complex, non-linear models like Random Forest. Unlike odds ratios, which measure association strength in more linear contexts, SHAP values allow us to understand both the direction and the magnitude of each feature’s impact on individual predictions within a tree-based model.

#### 3.2.2 Feature selection and contribution assessment using random forest

In this segment, we conducted an assessment of feature contribution using Random Forest and Shapley Values in combination with ADASYN and SMOTE (see Tables 6 and 7), evaluating both the importance of each feature and the direction of their influence on hypertension risk. Table 6 displays the results, detailing the RF Importance, average SHAP values, direction (positive or negative), and the associated impact (whether it increases or decreases hypertension risk). The direction and impact of each feature in predicting hypertension risk were determined through SHAP values, which offer insight into how each feature contributes to increasing or decreasing the model’s predictions.

**Table 6:** Feature contribution using RF Importance, SHAP Value and ADASYN.

Feature	RF Importance	Avg SHAP	Direction	Impact
BMI	2.7409	$8.67\times 10^{-19}$	Positive	Increases risk
Walk-mins-day-low	1.1688	$8.67\times 10^{-19}$	Positive	Increases risk
Hundred-cigarettes	1.2679	$8.67\times 10^{-19}$	Positive	Increases risk
Trait-anxiety-high	1.5454	$7.59\times 10^{-19}$	Positive	Increases risk
Sitting-mins-day-medium	1.5502	$4.34\times 10^{-19}$	Positive	Increases risk
College	1.0331	$3.79\times 10^{-19}$	Positive	Increases risk
Lack-air	3.2119	$3.25\times 10^{-19}$	Positive	Increases risk
Sitting-mins-weekend-high	1.1519	$3.25\times 10^{-19}$	Positive	Increases risk
Mother-with-hypertension	1.3509	$1.08\times 10^{-19}$	Positive	Increases risk
Smoking-father	0.8769	$6.78\times 10^{-21}$	Positive	Increases risk
Get-enough-sleep	1.3561	0	Negative	Decreases risk
Waist-size	1.5304	0	Negative	Decreases risk
Hoursasleep	1.2102	$-1.63\times 10^{-19}$	Negative	Decreases risk
Father-with-hypertension	2.0865	$-2.17\times 10^{-19}$	Negative	Decreases risk
Monocytes	1.3529	$-2.17\times 10^{-19}$	Negative	Decreases risk
Trait-anxiety-medium	1.3610	$-2.17\times 10^{-19}$	Negative	Decreases risk
Leukocytes	1.4257	$-4.34\times 10^{-19}$	Negative	Decreases risk
Erythrocytes	1.1437	$-4.88\times 10^{-19}$	Negative	Decreases risk
Triglycerides	1.8687	$-1.73\times 10^{-18}$	Negative	Decreases risk

**Table 7:** Feature contribution using RF Importance, SHAP Value and SMOTE.

Feature	RF_Importance	Avg SHAP	Efect	Impact
Lack-air	4.1963	$2.17\times 10^{-19}$	Positive	Increases risk
Trait-anxiety-high	1.3979	$1.08\times 10^{-18}$	Positive	Increases risk
Sitting-mins-day-medium	1.4569	$3.25\times 10^{-19}$	Positive	Increases risk
Physical-activity-moderate	0.7620	$3.25\times 10^{-19}$	Positive	Increases risk
Worker-qualify-professional	1.3819	$2.17\times 10^{-19}$	Positive	Increases risk
SS-Headache	0.7255	$3.05\times 10^{-20}$	Positive	Increases risk
Weight	1.5703	$5.42\times 10^{-20}$	Positive	Increases risk
Waist-size	1.5335	$6.51\times 10^{-19}$	Positive	Increases risk
BMI	2.3840	$-4.34\times 10^{-19}$	Negative	Decreases risk
College	1.2842	$-1.41\times 10^{-18}$	Negative	Decreases risk
Platelets	1.4541	$-1.30\times 10^{-18}$	Negative	Decreases risk
Physical-activity-low	0.5833	$-6.51\times 10^{-19}$	Negative	Decreases risk
Father-with-hypertension	1.3212	$-2.17\times 10^{-19}$	Negative	Decreases risk
MCH	1.3172	0	Negative	Decreases risk
Age	1.2112	$-4.34\times 10^{-19}$	Negative	Decreases risk
Snores	0.8557	$-6.78\times 10^{-21}$	Negative	Decreases risk
Peacefulsleep	0.7813	$-3.04\times 10^{-18}$	Negative	Decreases risk
MPV	1.3552	$-2.17\times 10^{-19}$	Negative	Decreases risk
HDL-cholesterol	1.1414	0	Negative	Decreases risk

Key variables that significantly increase hypertension risk included BMI, Lack-air, Hundred-cigarettes, Trait-anxiety-high and Mother-with-hypertension. These variables not only showed high RF importance values but also exhibit positive SHAP values, reinforcing their critical role in hypertension risk elevation.

Conversely, features such as Get-enough-sleep, Waist-size, and Father-with-hypertension were identified as non-contributory to the predictive model, potentially due to limited representation in the sample.

On the other hand, the analysis using SMOTE revealed significant features impacting hypertension risk, as shown in Table 7. Lack-air emerged as the most influential variable, with the highest RF importance score (4.1963) and a positive effect, indicating a strong association with increased hypertension risk. Trait-anxiety-high and Sitting-mins-day-medium also demonstrated high importance scores and positive SHAP values, highlighting the impact of anxiety-related and sedentary behaviors on hypertension.

Additional features, including Physical-activity-moderate, Worker-qualify-professional, and SS-Headache (Hours-fall-asleep and wake up with a headache), also displayed positive directions. Moreover, obesity-related factors such as weight and waist size showed significant positive impacts, emphasizing their well-documented link to cardiovascular health issues.

Other features, such as Age, Snores, and HDL-cholesterol, presented negative SHAP values and impacts showed non-risk associations. The use of SMOTE for balancing may have influenced these results by modifying feature distributions, potentially impacting traditional risk patterns. This highlights the importance of testing with various balancing methods, as was done here with both ADASYN and SMOTE, to better understand and validate feature impacts on hypertension prediction.

The results obtained with ADASYN and SMOTE as balancing techniques (see Table 5) showed clear distinctions in model performance. The ADASYN approach yielded a slightly higher balanced accuracy of 92%, with a sensitivity of 0.8647 and specificity of 0.9846, suggesting a stronger overall predictive power compared to SMOTE. The model using SMOTE, while slightly lower in balanced accuracy at 90%, still demonstrated high specificity (0.9945) and respectable sensitivity (0.8248). These results indicate that ADASYN may provide a slight advantage in identifying hypertension cases, while SMOTE excels in accurately identifying non-hypertension cases.

After identifying the most influential variables in the hypertension prediction model using Random Forest on the complete cohort, conducting a sex-specific analysis was deemed essential. Given that hypertension is influenced by multiple factors, some of which may vary substantially between men and women, a separate analysis allows for the identification of specific and relevant patterns within each group.

#### 3.2.3 Gender-specific feature analysis with random forest and shapley values

This section presents a stratified analysis to examine variables influencing hypertension risk separately in men and women subjects. This approach aims to reveal sex-specific risk factors that could remain hidden when analyzing the cohort as a whole. Unlike a matched analysis, which may obscure natural variations in risk by enforcing age and sex similarities, this stratified assessment respects the inherent differences within each subgroup, offering insights similar to those from a natural cohort.

To begin the gender-specific analysis, we conducted separate evaluations for men and women to examine how hypertension risk factors differ in influence across genders. Using sex-stratified Random Forest to identify key variables and SHAP values to assess each feature’s contribution, we applied ADASYN and SMOTE once more to address class imbalances in each subset.

**Figure 6:**
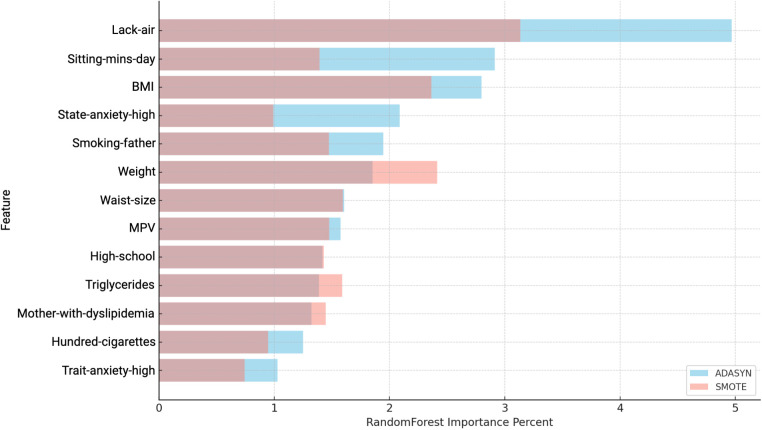
Comparison of Variable Importance by Random Forest using ADASYN and SMOTE in women. *Lack-air*, Waking up feeling short of breath and/or headache; *Sitting-mins-day*, Minutes sitting per day; *BMI*, Body mass index; *State-anxiety*, Anxiety as a state; *MPV*, Mean platelet volume; *Trait-anxiety*, Anxiety as a trait.

Figure 6 presents a comparison of variable importance using ADASYN and SMOTE in conjunction with Random Forest. Although Random Forest assigned high importance scores to variables such as Lack-air, Sitting-mins-day-high, and BMI, these rankings alone did not confirm each variable’s actual contribution to the prediction of hypertension risk. To better understand whether these variables indeed influence the model positively or negatively, and to what extent, SHAP values were used (see Tables 8 and 9).

**Table 8:** Feature contribution in women: Random Forest and SHAP Analysis Using ADASYN and SMOTE.

Method	Feature	RF_Imp_Per	Avg SHAP	Efect	Impact	Method	Feature	RF_Imp_Per	Avg SHAP	Efect	Impact
ADASYN	Father-with-diabetes	1.23204116	$8.67\times 10^{-19}$	Positive	Increases Risk	SMOTE	MCV	0.934081001	$1.08\times 10^{-18}$	Positive	Increases Risk
	Father-with-hypertension	1.61135937	$5.42\times 10^{-19}$	Positive	Increases Risk		Smoking-father	1.475593918	$8.67\times 10^{-19}$	Positive	Increases Risk
	Waist-size	1.60548896	$2.17\times 10^{-19}$	Positive	Increases Risk		BMI	2.363703052	$4.34\times 10^{-19}$	Positive	Increases Risk
	High	1.41817614	$1.08\times 10^{-19}$	Positive	Increases Risk		MPV	1.477161635	$4.34\times 10^{-19}$	Positive	Increases Risk
	Drink-alcohol	1.28678111	$1.08\times 10^{-19}$	Positive	Increases Risk		Weight	2.413789243	$2.17\times 10^{-19}$	Positive	Increases Risk
	Lack-air	4.96781438	0	Negative	Decreases Risk		Sitting-mins-day-high	1.393485218	$2.17\times 10^{-19}$	Positive	Increases Risk
	BMI	2.7986372	0	Negative	Decreases Risk		Hours-fall-asleep	1.126120744	$2.17\times 10^{-19}$	Positive	Increases Risk
	Trait-anxiety-high	2.08934303	0	Negative	Decreases Risk		Sitting-mins-weekend-medium	1.331035005	$2.17\times 10^{-19}$	Positive	Increases Risk
	Smoking-father	1.94476394	0	Negative	Decreases Risk		Triglycerides	1.588564846	$2.17\times 10^{-19}$	Positive	Increases Risk
	Sitting-mins-weekend-high	1.22197996	0	Negative	Decreases Risk		Trait-anxiety-high	0.992704097	$1.63\times 10^{-19}$	Positive	Increases Risk
	Worker-qualify-professional	1.11029064	$-2.75\times 10^{-20}$	Negative	Decreases Risk		Seric-iron	1.389980578	$1.08\times 10^{-19}$	Positive	Increases Risk
	MPV	1.57625213	$-5.42\times 10^{-20}$	Negative	Decreases Risk		Hundred-cigarettes	0.948250704	$1.08\times 10^{-19}$	Positive	Increases Risk
	Triglycerides	1.3875936	$-8.13\times 10^{-20}$	Negative	Decreases Risk		Waist-size	1.594882518	$1.69\times 10^{-21}$	Positive	Increases Risk
	Trait-anxiety-high	1.02850191	$-2.17\times 10^{-19}$	Negative	Decreases Risk		High	1.428547818	0	Negative	Decreases Risk
	Weight	1.85407959	$-2.71\times 10^{-19}$	Negative	Decreases Risk		Lack-air	3.135865093	$-5.42\times 10^{-20}$	Negative	Decreases Risk
	Mother-with-dyslipidemia	1.32249111	$-3.25\times 10^{-19}$	Negative	Decreases Risk		RDW	1.630786454	$-1.08\times 10^{-19}$	Negative	Decreases Risk
	Hundred-cigarettes	1.25021331	$-4.34\times 10^{-19}$	Negative	Decreases Risk		Atherogenic-index	1.279079458	$-1.63\times 10^{-19}$	Negative	Decreases Risk
	LymphocytesN	1.88576733	$-8.67\times 10^{-19}$	Negative	Decreases Risk		Trait-anxiety-high	0.741266069	$-4.34\times 10^{-19}$	Negative	Decreases Risk
	Sitting-mins-day-high	2.91392131	$-1.08\times 10^{-18}$	Negative	Decreases Risk		Take-naps	1.365467715	$-4.34\times 10^{-19}$	Negative	Decreases Risk
	State-anxiety-medium	1.09661153	$-1.73\times 10^{-18}$	Negative	Decreases Risk		Mother-with-dyslipidemia	1.446227987	$-8.67\times 10^{-19}$	Negative	Decreases Risk

**Table 9:** Feature contribution in men: Random Forest and SHAP Analysis using ADASYN and SMOTE.

Method	Feature	RF_Imp_Per	Avg SHAP	Efect	Impact	Method	Feature	RF_Imp_Per	Avg SHAP	Efect	Impact
ADASYN	Waist-size	2.88050419	$1.73\times 10^{-18}$	Positive	Increases risk	SMOTE	Weight	2.1682248	$4.34\times 10^{-18}$	Positive	Increases risk
	Total-cholesterol	2.58451633	$1.73\times 10^{-18}$	Positive	Increases risk		NeutrophilsN	1.81985804	$1.52\times 10^{-18}$	Positive	Increases risk
	Optimal-sleep	1.48741887	$1.73\times 10^{-18}$	Positive	Increases risk		Lack-air	2.73009092	$1.30\times 10^{-18}$	Positive	Increases risk
	High	2.45519573	$1.08\times 10^{-18}$	Positive	Increases risk		Mother-with-obesity	1.67421861	$1.08\times 10^{-18}$	Positive	Increases risk
	Leukocytes	1.76374815	$9.22\times 10^{-19}$	Positive	Increases risk		Eosinophils	1.86409221	$4.34\times 10^{-19}$	Positive	Increases risk
	HDL-cholesterol	2.74866834	$6.51\times 10^{-19}$	Positive	Increases risk		Sitting-mins-weekend-low	1.01886438	$3.25\times 10^{-19}$	Positive	Increases risk
	Trait-anxiety-high	0.50669197	$4.34\times 10^{-19}$	Positive	Increases risk		Mother-with-hypertension	1.03589995	$3.25\times 10^{-19}$	Positive	Increases risk
	Eosinophils (%)	2.81581707	$2.17\times 10^{-19}$	Positive	Increases risk		BMI	3.19687327	$2.17\times 10^{-19}$	Positive	Increases risk
	Heart-rate	1.426956	$1.36\times 10^{-19}$	Positive	Increases risk		Trait-anxiety-high	1.18264605	$2.17\times 10^{-19}$	Positive	Increases risk
	Weight	2.87057579	$5.42\times 10^{-20}$	Positive	Increases risk		Total-cholesterol	1.83283462	$1.08\times 10^{-19}$	Positive	Increases risk
	Get-enough-sleep	1.60738549	$2.71\times 10^{-20}$	Positive	Increases risk		Trait-anxiety-high	1.08616841	$1.08\times 10^{-19}$	Positive	Increases risk
	Lack-air	1.63956115	0	Negative	Decreases risk		Triglycerides	2.06711864	$5.42\times 10^{-20}$	Positive	Increases risk
	Snores	1.42475929	0	Negative	Decreases risk		Serumsodium	1.47789595	$3.39\times 10^{-20}$	Positive	Increases risk
	Father-with-hypertension	1.9096533	0	Negative	Decreases risk		Eosinophils (%)	1.75537788	0	Negative	Decreases risk
	Smoking-mother	0.43988346	0	Negative	Decreases risk		Snores	1.70658864	0	Negative	Decreases risk
	Mother-with-hypertension	1.0297687	$-1.63\times 10^{-19}$	Negative	Decreases risk		HDL-cholesterol	1.44923223	$-1.08\times 10^{-19}$	Negative	Decreases risk
	Trait-anxiety-high	2.0744688	$-3.25\times 10^{-19}$	Negative	Decreases risk		Waist-size	2.15200906	$-4.34\times 10^{-19}$	Negative	Decreases risk
	BMI	3.13136316	$-5.42\times 10^{-19}$	Negative	Decreases risk		Hours-fall-asleep	1.43852531	$-4.34\times 10^{-19}$	Negative	Decreases risk
	SDI-level	0.70075627	$-9.76\times 10^{-19}$	Negative	Decreases risk		Heart-rate	1.29510572	$-6.51\times 10^{-19}$	Negative	Decreases risk
	Eosinophils	2.21694876	$-3.90\times 10^{-18}$	Negative	Decreases risk		SDI-level	1.14577519	$-8.67\times 10^{-19}$	Negative	Decreases risk

The analysis for women using ADASYN highlights specific features with a strong impact on hypertension risk. Factors like Father-with-diabetes, Father-with-hypertension, Waist-size, High-school and Drink-alcohol were prominent, with positive SHAP values and a high Random Forest importance percentage, indicating an increased risk for hypertension.

In this analysis of feature contribution for women (see Table 8), we observe distinct differences between the results from ADASYN and SMOTE. SMOTE identified a broader set of risk factors associated with hypertension in the women sample. Key features like MCV, BMI, MPV, and Triglycerides exhibit a high importance score, suggesting a strong association with increased hypertension risk. These variables have positive SHAP values, reinforcing their role as significant risk contributors.

Additional features highlighted by SMOTE include lifestyle and demographic factors, such as Smoking-father, Hundred-cigarettes, Sitting-mins-day-high, Sitting-mins-weekend-medium and State-anxiety-high, which also show positive SHAP values, indicating an increase in hypertension risk. This expanded set of risk factors identified by SMOTE may provide a more nuanced understanding of the variables impacting hypertension in women, suggesting that SMOTE’s sampling method could be particularly effective in detecting a wider range of risk factors in this population.

**Figure 7:**
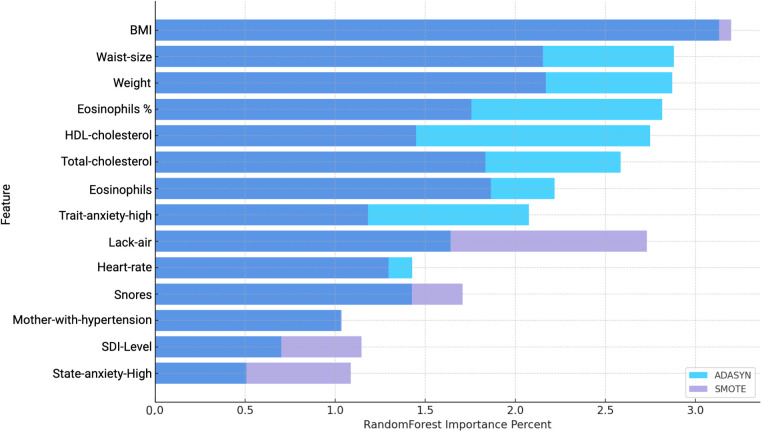
Comparison of Variable Importance by Random Forest using ADASYN and SMOTE in men. *BMI*, Body mass index; *Waist-size*, Waist circumference; *Trait-anxiety*, Anxiety as a trait; *Lack-air*, Waking up feeling short of breath and/or headache; *SDI-Level*, Social Development Index; *State-anxiety*, Anxiety as a state.

In the analysis of men participants, using both ADASYN and SMOTE balancing techniques with Random Forest revealed a range of features relevant to hypertension prediction. As shown in Figure 7 and Table 9, several variables demonstrated high importance scores in the Random Forest model, with ADASYN and SMOTE highlighting factors such as BMI, Waist-size, Weight, Eosinophils, Total-cholesterol and Lack-air as having substantial influence.

However, despite the high importance scores observed in the Random Forest Importance Percent (see Figure 7), these variables do not necessarily exhibit strong predictive contributions according to the average SHAP values (see Table 9). SHAP analysis provides a nuanced view by assessing the actual impact and direction of these features on hypertension risk.

In the ADASYN-based analysis for men (see Table 9), focusing on the average SHAP values features such as waist size, total cholesterol, Optimal-sleep, High-school, State-anxiety-high and Weight suggest these factors strongly increase hypertension risk. Lower-ranking features with negative SHAP values, like SDI-Level and Trait-anxiety High, suggest a reduced association with hypertension risk in this men cohort when adjusted through ADASYN.

In the SMOTE-based analysis for men, several features stood out with high average SHAP values, indicating their significant influence on hypertension risk. Weight, Neutrophils and Lack-air had positive SHAP values and high importance scores, suggesting they strongly contribute to the likelihood of hypertension in this population. The high SHAP values for Neutrophils and Eosinophils are notable, as they point to inflammatory markers being potential risk factors for hypertension in men, aligning with research that associates inflammation with cardiovascular conditions.

Mother-with-obesity and Mother-with-hypertension also exhibit positive SHAP values, highlighting familial and genetic influences on hypertension risk. Additionally, BMI, total cholesterol, and Trait-anxiety-high reflect the expected positive impact on hypertension, underscoring the relevance of body composition, cholesterol, and stress-related factors in hypertension development. Interestingly, SDI-level also shows a negative SHAP value, indicating a possible association between socioeconomic status and lower hypertension risk in men. This nuanced insight highlights how socioeconomic factors might play a role in cardiovascular health, possibly through better access to resources or health-promoting behaviors.

The results for men and women using the SMOTE technique showed a significant performance distinction based on the balanced accuracy metric. For women, the model with SMOTE achieved a balanced accuracy of 90%, highlighting strong predictive performance and stability across sensitivity and specificity. For men, however, the SMOTE technique yielded an even higher balanced accuracy of 92% under a sensibility of 0.8414 and an specificity of 0.9814 indicating that the model performed exceptionally well with a simpler configuration (see Table 5. This highlights SMOTE’s adaptability in adjusting to gender-specific data nuances, enhancing hypertension prediction for both men and women while capturing distinctive data patterns within each subgroup.

**Figure 8:**
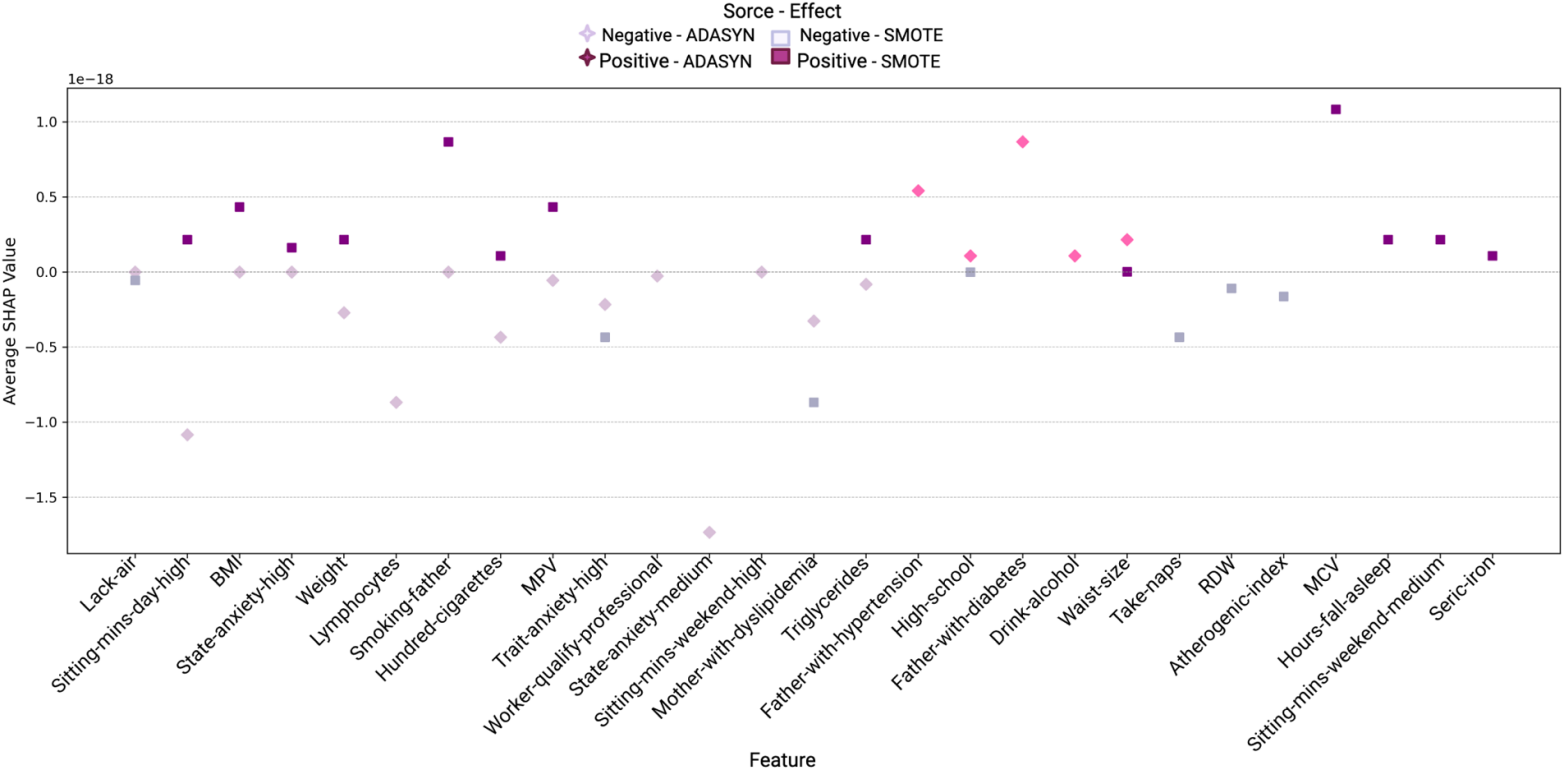
Variable impact on women using SHAP with Random Forest and ADASYN and SMOTE methods.

**Figure 9:**
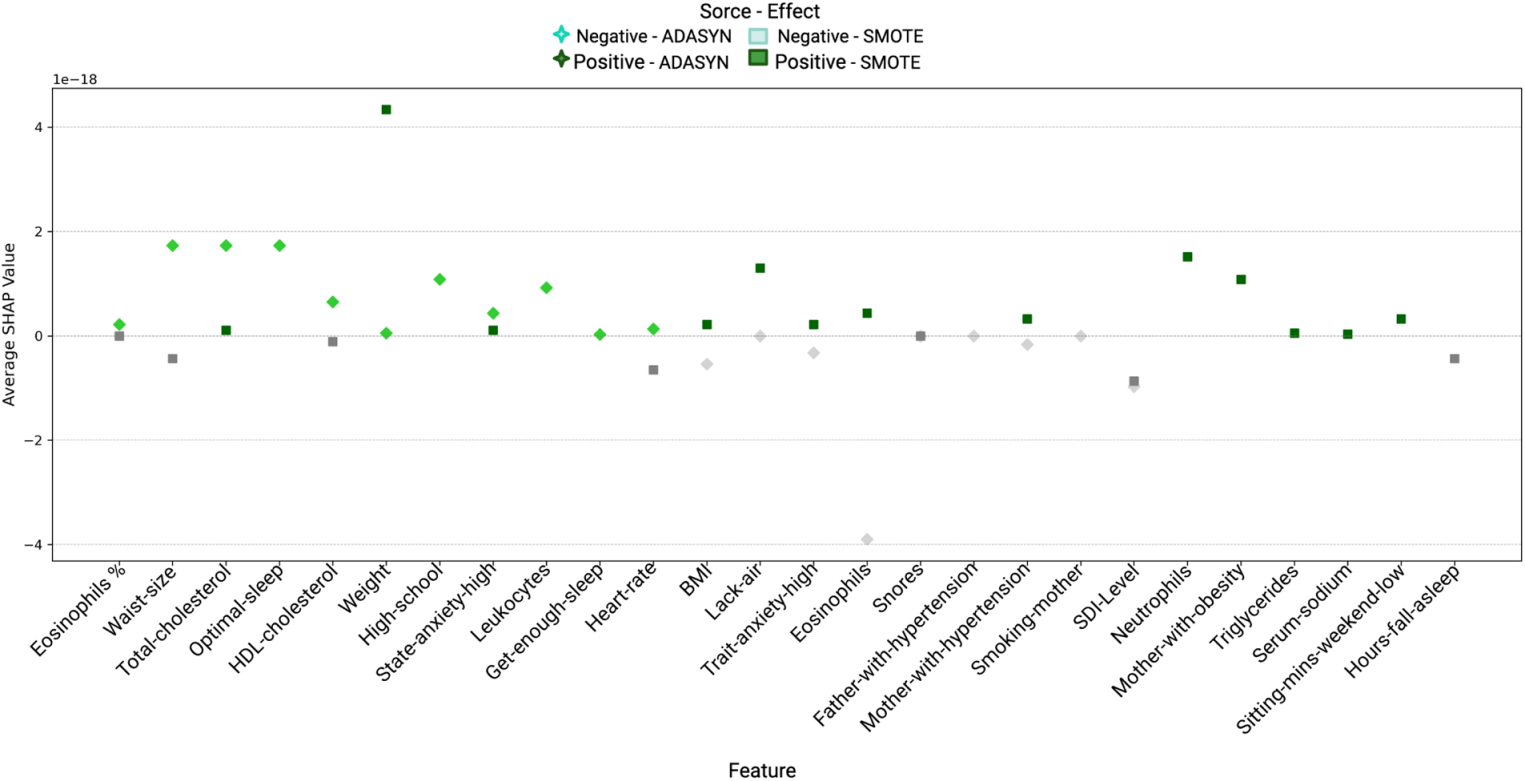
Variable impact on men using SHAP with Random Forest and ADASYN and SMOTE methods.

Figures 8 and 9 display the SHAP values for women and men, respectively, highlighting the impact of various features on hypertension prediction within each gender. Each point represents a feature’s average SHAP value, indicating its contribution direction and intensity to the model. Positive values suggest an increase in hypertension risk, while negative values imply a protective effect. The color and shape coding differentiates the effects under ADASYN (green for positive and red for negative) and SMOTE (orange for positive and blue for negative), allowing a comparison of the balancing methods’ influence on variable impacts. These visualizations facilitate a clearer understanding of how certain features uniquely contribute to the model’s predictions for each gender, capturing both risk-increasing and risk-reducing variables.

## 4 Discussion

Hypertension has been widely recognized as a multifaceted condition influenced by an array of genetic, environmental, and lifestyle factors, with age and sex being two well-documented determinants. In this study, we applied machine learning algorithms to investigate additional risk factors in a relatively young cohort (under 50 years old) that is otherwise healthy, drawn from a metropolitan population in México City. The choice of XGBoost, SVM and RF models for this analysis stems from their proven effectiveness in similar research, particularly in developing predictive models for hypertension and cardiovascular diseases (46, 47). Additionally, we employed SHAP approach to assess the significance of each variable in the dataset. In the context of this study, it aids in feature selection by quantifying the impact of each variable on the model’s performance, thereby enabling the construction of the most efficient and accurate predictive model.

Building upon the foundational data in Tables 1, 2, 3, 4, 6 and 7, and in light of the superior performance metrics presented in Table 5, our findings extend beyond well-known hypertension risk factors such as weight and elevated levels of sodium, LDL-cholesterol, or triglycerides. The analysis uncovers a spectrum of significant contributors to hypertension, encompassing sleep disturbances, dietary habits, and lifestyle attributes characterized by diminished physical activity and sedentary behaviors. Additionally, habitual consumption of energy drinks and alcoholic beverages, coupled with socio-economic factors like education and employment status, emerged as pertinent influences of hypertension risk.

Sleep disorders –in particular obstructive sleep apnea– have been recently associated with hypertension in young adults (48–50) and even in children (51). The evidence however is still weak. It has been indeed highlighted that in order to fully establish this relationship, well-designed disease-driven, interventional studies and even powered controlled trials are needed (52). These studies are actually gradually appearing (53–55). In this regard, though the present study has not the scope highlighted by Van Ryswyk and collaborators (52), it is indeed along the same lines regarding the use of systematic cohort studies and controlling for confounding variables such as sex and years. Furthermore, since our study was not driven by any particular hypothesis in this concern and since a large number of other potentially associated features were considered, it contributes to the (gradual) establishment of this association.

Physiology-based studies have also pointed out to a possible relationship between sleep disorders and cardiovascular diseases, in general (56). Sympathetic stimulation, oxidative stress, and systemic inflammation constitute the primary intermediary mechanisms that are presumably linked to sleep apnea and intermittent hypoxia. Accumulating evidence supports the connections between hypertension, cardiac arrhythmias, stroke, coronary heart disease, and heightened cardiovascular mortality in individuals affected by these conditions. One particular area of interest has been the cardio-respiratory phenomena present at the onset of sleep. The commencement of sleep brings about significant cardio-respiratory alterations. These changes vary depending on the sleep stage, leading to distinct hemodynamic and autonomic responses. For instance, in non-REM sleep, there is a notable decline in heart rate, systolic blood pressure, and cardiac output by as much as 15%. These modifications are most pronounced during slow wave sleep and are believed to be attributed to shifts in autonomic activity (57).

The role of diet significantly influences the risk of cardiovascular disease, with nutrients playing a crucial role in mitigating hypertension (58). Noteworthy the effects of SFAs, PUFAs, and MUFAs, are closely linked to hypertension but is unclear (59). Dietary intake of SFAs and some of their subtypes (*C6:0*, *C14:0*, *C16:0*, and *C18:0*) are acknowledged as a possible risk factor for hypertension, particularly in middle-aged adults. Nevertheless, certain research has indicated that the consumption of SFAs (*C4:0*, *C6:0*, *C12:0*, *C14:0*, and *C16:0*) may be associated with a reduced likelihood of developing hypertension in the elderly. The relationship between the dietary consumption of SFAs and its subcategories and hypertension has been a less explored area in prior research (60).

It is widely recognized that incorporating unsaturated fatty acids (PUFAs, and MUFAs) into daily diet reduces the chances of hypertension and cardiovascular disease by alleviating inflammation and oxidative stress in vascular endothelial tissues (61). MUFAs enhance the antioxidant capacity and receptor activity of low-density lipoprotein, facilitating the removal of low-density lipoprotein from circulation. This, in turn, safeguards the vascular endothelium and serves as a preventive measure against cardiovascular diseases (59).

Some studies have demonstrated the protective impact of a Mediterranean diet, characterized by increased intake of MUFAs on overall BP management. However, this association was not observed in trials exclusively involving normotensive subjects. Additional research is required to elucidate the potential impact of diets rich in MUFAs on BP reduction (62). Polyunsaturated fatty acids contribute to a lower occurrence of coronary artery disease by decreasing plasma levels of low-density lipoprotein cholesterol and very low-density lipoprotein cholesterol, while simultaneously increasing high-density lipoprotein cholesterol. Specifically, omega-3 polyunsaturated fatty acids can impact various factors, including cholesterol levels, fat cell metabolism, adipogenesis, inflammation, thrombosis, and atherosclerosis, thereby reducing the risk of cardiovascular disease (59).

Our results also found an important relation of vitamins such as Riboflavin (*B12*) and (*Folic-acid*) as well as Tocopherols (γ-*Tocopherol*) and some minerals (*Sodium*, *Calcium*) related with hypertension. Regarding some elements like potassium, niacin, thiamine, riboflavin, vitamin B12, iron, and magnesium have been identified as substances that can lower BP (62). Several studies have proposed that vitamin consumption plays a role in regulating BP. Dietary patterns rich in fruits, vegetables, and antioxidants are suggested to reduce the risk of hypertension (*HTN*). Fruits and vegetables, high in potassium, provide cardiovascular protection by countering sodium effects through enhanced urinary excretion and vasodilation. Magnesium, a cofactor for enzymes in vascular signaling pathways, inhibits vasoconstriction and correlates with improved hemodynamic status. Magnesium is also beneficial in preventing HTN by mitigating oxidative stress damage to the vasculature (62).

Additional features related to anxiety and sleep disorder were also found out, as reported in Tables 1 and 2. For instance, in connection with the link between anxiety and hypertension, it has also been argued that such a relationship may have physiological (and often bidirectional) origins. In brief, psychosocial stressors linked to anxiety disorders may be able to elevate autonomic arousal by activating the hypothalamic-pituitary axis, leading to heightened levels of circulating catecholamines. This heightened state of arousal has been linked to an elevated risk of hypertension and the promotion of a pro-inflammatory environment, ultimately contributing to the development of coronary heart disease. Importantly, this association persists across various anxiety disorders, including generalized anxiety, post-traumatic stress disorder, panic disorder, and obsessive-compulsive disorder. Furthermore, the connection remains significant even when accounting for comorbid conditions such as depression and physical health issues (63).

Recent longitudinal and cross-sectional investigations conducted across various geographical regions and age brackets consistently reveal a strong link between coexisting anxiety and both existing and newly diagnosed cases of hypertension. The expanding body of research on blood pressure fluctuations and diminished baroreflex sensitivity, stemming from autonomic irregularities, offers deeper insights into the underlying mechanisms connecting anxiety and hypertension (64).

Observational studies indicate that young adults, upon receiving an anxiety diagnosis, face an elevated risk of developing hypertension, suggesting prolonged exposure to disruptions in autonomic functions. Furthermore, the complex interplay of joint anxiety and depression may contribute to previous conflicting findings regarding the association between anxiety and hypertension (65). In this context, our findings contribute to ascertain this relationship, in particular for the case considered of relatively young adults in a urban environment. Interestingly, most of the studies on the relationship between anxiety and hypertension in urban environments, refer in some way to hypertension-driven anxiety rather than anxiety-driven onset of hypertension (66). This is relevant, since all participants in our study cohort were hypertension-free at the baseline (hypertension was indeed an exclusion criteria on recruitment), so anxiety traits in the participants are not driven by hypertension but can nevertheless be linked to a third unobserved effect.

Unlike the associations of hypertension with sleep disorders and anxiety, that although not yet established have been previously documented and analyzed; we have unveiled another statistically significant association that at the moment has been just marginally described: energy drink consumption. The association of energy drink consumption with cardiovascular outcomes has been slowly emerging from anecdotal (67) to plausibility arguments related to mixed effects with concomitant alcohol consumption (68) and dosage (69) to small *acute* effects on cardiovascular indicators such as temporary increased heart rate and SBP (below the hypertension regime) in small controlled trials (70). No conclusive association has yet established (71). Indeed the current literature on the issue talks about *products that may cause an increase in blood pressure* (72) or *potential risks for young adults* (73). Given the relatively large, sustained effect that we have found, it becomes worthy to plan for targeted analysis, such as randomized intervention trails and systematic cohort studies to evaluate whether this association is indeed causal or there are some hidden or confounding variables (16, 74). Along the same lines, it may also be advantageous to investigate on the potential molecular and physiological origins in this association, perhaps in animal models (75, 76).

The study also incorporates a sex-stratified analysis to reveal gender-specific risk factors. For example, women showed significant associations with parental hypertension and obesity, whereas in men, lifestyle factors like physical inactivity and stress were prominent. These distinctions highlight how social and biological determinants interact differently across genders. Variables such as sleep disorders, sedentary behaviors, anxiety levels, and specific dietary habits were identified as significant contributors to hypertension. The interplay of these modifiable factors can provide targeted prevention strategies for men and women.

The use of advanced machine learning techniques, including XGBoost and SHAP, enhances the ability to uncover non-linear and complex relationships within health data. This allows for a nuanced understanding of how combinations of variables contribute to hypertension risk, going beyond traditional linear models. The machine learning models achieved high predictive accuracies (balanced accuracy up to 92% using Random Forest and ADASYN). These tools are effective for identifying at-risk individuals early and tailoring interventions.

SHAP analysis provided insights into the relative importance of risk factors and their specific contributions to hypertension. For instance, “minutes sitting per day” and “BMI” emerged as key predictors with consistent impacts on hypertension risk. Techniques like SMOTE and ADASYN addressed data imbalance, ensuring reliable model performance and equitable learning from minority and majority classes.

The findings of this study are highly relevant for distinguishing differences in emerging risk factors for arterial hypertension between men and women. The application of machine learning not only strengthens the predictive modeling of these risk factors but also allows for actionable insights to tailor gender-specific preventive and management strategies.

### 4.1 Limitations

This research was based on data obtained from a cohort of relatively healthy adult residents of México City and does not represent the country. New-onset hypertension was defined as the condition of a previously normotensive participant who had hypertension (either blood pressures ≥140/≥90 mm Hg or the use of an anti-hypertensive medication) only in one attended office examination at Instituto Nacional de Cardiología Ignacio Chávez. For this study, we chose the pre-established cutoff point of the original protocol (≥140/≥90 mm Hg) recommended in the JNC 7 report (33). However, we may have underestimated those cases with hypertension now defined with SBP of 130–139 or a DBP of 80–89 mm Hg accordingly the most recent international guidance (77).

## 5 Conclusions and perspectives

In conclusion, our study in a relatively young and healthy cohort from México City has shed light on the early impact of risk factors for hypertension beyond the well-established contributors of age and weight. Notably, we found significant associations between hypertension and sleep disorders, consumption of certain nutrients and regular consumption of energy drinks as well as some lifestyle characteristics. While the evidence connecting sleep disorders, particularly obstructive sleep apnea, to hypertension is still evolving, our study adds to the growing body of research supporting this relationship. Moreover, we highlighted the potential physiological mechanisms linking sleep disorders to cardiovascular diseases, emphasizing the importance of further investigations into this complex interplay.

The association between anxiety and hypertension, which appears bidirectional and persists across various anxiety disorders, has been reinforced by our findings. Importantly, our study focused on relatively young adults in an urban environment and underscores the need to explore this relationship in different demographic groups. Interestingly, our cohort was hypertension-free at baseline, emphasizing that anxiety traits may contribute to hypertension independently, offering valuable insights for preventive strategies.

One of the most striking findings of our study is the statistically significant association between energy drink consumption and hypertension risk. The substantial increase in hypertension risk associated with energy drink consumption warrants further investigation, including randomized intervention trials and systematic cohort studies, to determine causality and identify potential confounding variables. Overall, our study contributes to our understanding of hypertension risk factors, highlighting the importance of addressing sleep disorders, anxiety, and energy drink consumption in hypertension prevention and management strategies.

The application of machine learning techniques, including XGBoost, SVM, and Random Forest, provided a robust framework for identifying hypertension risk factors in this study. Using SHAP values, along with balancing techniques like ADASYN and SMOTE, we evaluated not only the importance of specific variables but also their direction and contribution to hypertension risk.

XGBoost and SVM models allowed for initial feature selection, with Random Forest further refining the analysis. SHAP values offered critical insights into each feature’s influence, while ADASYN and SMOTE highlighted gender-specific patterns, with SMOTE uncovering a broader array of risk factors in women. Importantly, SHAP analysis showed that some variables identified by Random Forest as important do not necessarily increase hypertension risk, underscoring the need for interpretative tools to clarify complex relationships.

## Data Availability

The original contributions presented in the study are included in the article/supplementary material, further inquiries can be directed to the corresponding author/s.
