# Supplementary material for: Machine learning analysis of emerging risk factors for early-onset hypertension in the Tlalpan 2020 cohort
Source: Front Cardiovasc Med. 2025 Jan 17;11:1434418. doi: 10.3389/fcvm.2024.1434418 (PMC11782138; doi:10.3389/fcvm.2024.1434418)
Supplement: Supplementary file 1 [file Datasheet1.pdf]

Machine Learning Analysis of Emerging Risk Factors for Early-onset Hypertension in the Tlalpan 2020 Cohort (Supplementary Materials)

Mireya Martínez-García [1,†]*, Guadalupe O. Gutiérrez-Esparza [2,3,†], Manlio F. Márquez [4], Luis M. Amezcua-Guerra [1], Enrique Hernández-Lemus [5,6]*

[1] *Department of Immunology, Instituto Nacional de Cardiología Ignacio Chávez, México City, México*
[2] *Investigadora por México CONAHCYT Consejo Nacional de Humanidades, Ciencias y Tecnologías, México City, México*
[3] *Instituto Nacional de Cardiología Ignacio Chávez, México City, México*
[4] *Department of Electrocardiology, Instituto Nacional de Cardiología Ignacio Chávez, México City, México*
[5] *Computational Genomics Division, Instituto Nacional de Medicina Genómica, México City, México*
[6] *Center for Complexity Sciences, Universidad Nacional Autónoma de México, México City, México*

Correspondence*:
Enrique Hernández-Lemus; Mireya Martínez-García
ehernandez@inmegen.gob.mx; mireya.martinez@cardiologia.org.mx

SUPPLEMENTARY TABLES 1 - 12

The following tables (labeled here as Tables 1 to 12) correspond to **Supplementary Table 1** to **Supplementary Table 12** respectively in the main manuscript.

Table 1. Anthropometric and risk factors among to hypertension and normotension participants.

Characteristics	Hypertension (150)	Normotension (600)	*P value* [a]
**Sex** [b]			
FeMen	92 (61.33)	368 (61.33)	–
Men	58 (38.67)	232 (38.67)	–
**Age (Years)** [c]			
Women	50 (47-55)	51 (47-55)	–
Men	48 (42-53)	48 (43-53)	–
**BMI (Kg/m^2)**			
Women	28.46 (25.97-34.22)	26.59 (23.80-29.72)	< 0.0001
Men	28.46 (26.83-31.16)	26.91 (24.21-29.92)	0.0019
**Weight (Kg)** [c]			
Women	70.30 (62.25-84.95)	65.65 (58.10-73.85)	< 0.0001
Men	83.05 (78.00-89.40)	78.50 (69.20-85.45)	0.0017
**Waist size (cm)**			
Women	92.50 (84.25-103.50)	88.00 (82.00-95.00)	0.0017
Men	98.00 (92.50-101.00)	94.60 (87.50-102.00)	0.0120
**Smoking (yes)** [b]			
Women	48 (32.00)	179 (29.88)	0.6765
Men	48 (32.00)	176 (29.38)	0.5902
**Smoking 100 (yes)** [b]			
Women	31 (31.96)	98 (27.61)	0.2556
Men	38 (39.18)	142 (40.00)	0.7485
**Former smoker (yes)** [b]			
Women	19 (19.59)	48 (13.56)	0.1026
Men	16 (16.49)	64 (18.08)	0.9999
**Passive smoker (yes)** [b]			
Women	22 (14.67)	80 (13.36)	0.7696
Men	21 (14.00)	57 (9.52)	0.1428
**Drink alcohol (Yes)** [b]			
Women	43 (28.67)	192 (32.05)	0.4909
Men	43 (28.67)	190 (31.72)	0.5409
**Drink energy drinks (Yes)** [b]			
Women	3 (2.10)	14 (2.36)	0.9999
Men	3 (2.10)	32 (5.39)	0.1298

[a] *P values* were obtained using Pearson's chi-squared test or Wilcoxon test accordingly.
[b] The values are expressed in n (%) and [c] med (IQR 25-75). *BMI*, body mass index

Table 2. Sociodemographic characteristics among to hypertension and sex and age matched normotension participants

**Characteristics**	**Hypertension (150)**	**Normotension (600)**	***P value*** [a]
**Educational level** [b]			
Elementary school			
Women	17 (11.33)	48 (8.01)	0.2561
Men	11 (7.33)	31 (5.18)	0.4044
High school			
Women	36 (24.00)	137 (22.87)	0.8454
Men	24 (16.00)	63 (10.52)	0.0821
College			
Women	31 (20.67)	138 (23.04)	0.6153
Men	20 (13.33)	100 (16.69)	0.3835
Postgraduate			
Women	8 (5.33)	45 (7.51)	0.4544
Men	3 (2.00)	37 (6.18)	0.0675
**Occupational class** [b]			
Student			
Women	1 (0.67)	5 (0.83)	0.9999
Men	1 (0.67)	6 (1.00)	0.9999
Business executive			
Women	11 (7.33)	45 (7.51)	0.9999
Men	5 (3.33)	15 (2.50)	0.7769
Housekeeper			
Women	25 (16.67)	95 (15.86)	0.9009
Men	0 (0)	1 (0.17)	0.9999
Worker qualify or Professional			
Women	42 (28.00)	177 (29.55)	0.7941
Men	41 (27.33)	165 (27.55)	0.9999
Worker unqualified			
Women	13 (8.67)	43 (7.18)	0.6516
Men	10 (6.67)	44 (7.35)	0.9156
Unemployed			
Women	0 (0)	3 (0.50)	0.8850
Men	1 (0.67)	0 (0)	0.4530
**Social Development Index** [b]			
Very low			
Women	11 (7.33)	52 (8.67)	0.7173
Men	8 (5.33)	25 (4.17)	0.6887
Low			
Women	36 (24.00)	130 (21.67)	0.6130
Men	23 (15.33)	74 (12.33)	0.3991
Medium			
Women	18 (12.00)	90 (15.00)	0.4202
Men	11 (7.33)	52 (8.67)	0.7173
High			
Women	27 (18.00)	96 (16.00)	0.6395
Men	16 (10.67)	81 (13.50)	0.4302

[a] *P values* were obtained using Pearson's chi-squared test or Wilcoxon test accordingly.
[b] The values are expressed in n (%) or [c] med (IQR 25-75) accordingly.

Table 3. Family background characteristics among to hypertension and sex and age matched normotension participant

Characteristics	Hypertension (150)	Normotension (600)	*P value* [a]
**Family background** [b]			
**Mother with obesity (Yes)**			
Women	28 (18.67)	112 (18.70)	0.9999
Men	12 (8.00)	60 (10.02)	0.5560
**Father with obesity (Yes)**			
Women	18 (12.00)	58 (9.68)	0.4866
Men	13 (8.67)	48 (8.01)	0.9202
**Mother smoking (Yes)**			
Women	10 (6.67)	36 (6.01)	0.9091
Men	12 (8.00)	35 (5.84)	0.4290
**Father smoking (Yes)**			
Women	25 (16.67)	115 (19.20)	0.5581
Men	19 (12.67)	78 (13.02)	0.9999
**Mother with diabetes (Yes)**			
Women	28 (18.67)	106 (17.70)	0.8675
Men	13 (8.67)	41 (6.84)	0.5483
**Father with diabetes (Yes)**			
Women	32 (21.33)	105 (17.53)	0.3327
Men	12 (8.00)	47 (7.85)	0.9999
**Mother with hypertension (Yes)**			
Women	42 (28.00)	170 (28.38)	0.9999
Men	22 (14.67)	69 (11.52)	0.3562
**Father with hypertension (Yes)**			
Women	29 (19.33)	108 (18.03)	0.7950
Men	19 (12.67)	46 (7.68)	0.0743
**Mother with dyslipidemia (Yes)**			
Women	22 (14.67)	107 (17.86)	0.4247
Men	12 (8.00)	48 (8.01)	0.9999
**Father with dyslipidemia (Yes)**			
Women	19 (12.67)	80 (13.36)	0.9355
Men	13 (8.67)	52 (8.68)	0.9999

[a] *P values* were obtained using Pearson's chi-squared test. [b] The values are expressed in n (%).

Table 4. Physical activity and psychological stress characteristics among to hypertension and sex and age matched normotension participant

Characteristics	Hypertension (150)	Normotension (600)	*P value* [a]
**Physical activity** [b]			
**Low**			
Women	11 (7.33)	51 (8.50)	0.7654
Men	11 (7.33)	38 (6.33)	0.7960
**Moderate**			
Women	41 (27.33)	172 (28.67)	0.8238
Men	23 (15.33)	96 (16.00)	0.9403
**High**			
Women	40 (26.67)	145 (24.17)	0.5965
Men	24 (16.00)	98 (16.33)	0.9999
**Vigorous activities (No)** [b]			
Women	81 (54.00)	324 (54.00)	0.9999
Men	49 (32.67)	190 (31.67)	0.8909
**Moderate activities (No)** [b]			
Women	79 (52.67)	313 (52.17)	0.9854
Men	44 (29.33)	181 (30.17)	0.9207
**Walk (No)** [b]			
Women	63 (42.00)	254 (42.33)	0.9999
Men	33 (22.00)	124 (20.67)	0.8050
**Walk (Hours of the day)** [c]			
Women	0 (0-15)	0 (0-10)	0.6166
Men	0 (0-30)	0 (0-30)	0.9807
**Sitting (Hours of the day)** [c]			
Women	300 (180-420)	240 (120-360)	0.0939
Men	300 (180-480)	240 (180-480)	0.7343
**Sitting (Hours of the weekend)** [c]			
Women	240 (150-300)	240 (120-300)	0.5529
Men	240 (120-300)	240 (180-330)	0.2176
**Psychological stress** [b]			
**Low**			
Women	60 (40)	262 (43.67)	0.4720
Men	46 (30.67)	172 (28.67)	0.7025
**Moderate**			
Women	30 (20.00)	96 (16.00)	0.2937
Men	11 (7.33)	57 (9.50)	0.5044
**Severe**			
Women	2 (1.33)	10 (1.67)	0.9999
Men	1 (0.67)	3 (0.5)	0.9999

[a] *P values* were obtained using Pearson's chi-squared test or Wilcoxon test accordingly.
[b] The values are expressed in n (%) and [c] med (IQR 25-75).

Table 5. Distribution of clinical parameters among to hypertension and sex and age matched normotension participant

**Characteristics**	**Hypertension (150)**	**Normotension (600)**	***P value*** [a]
**Total cholesterol (mg/dl)**			
Women	188.35 (172.80-209.80)	195.50 (173.15-216.45)	0.2437
Men	196.60 (167.90-214.00)	190.25 (164.40-224.25)	0.9008
**HDL cholesterol (mg/dl)**			
Women	47.00 (40.65-53.60)	49.35 (42.70-57.80)	0.0359
Men	38.85 (34.50-43.50)	41.50 (36.25-48.25)	0.0118
**LDL cholesterol (mg/dl)**			
Women	119.36 (104.45-137.05)	122.55 (104.20-139.95)	0.4603
Men	122.31 (102.40-143.20)	124.20 (100.05-144.70)	0.7660
**Atherogenic index** [c]			
Women	2.62 (2.14-3.07)	2.46 (1.94-3.08)	0.3483
Men	3.09 (2.68-3.67)	2.99 (2.44-3.46)	0.1816
**Triglycerides (mg/dl)**			
Women	149.85 (112.20-190.60)	138.60 (99.65-187.95)	0.0955
Men	194.85 (139.80-269.50)	153.90 (113.85-225.5)	0.0077
**Glucose (mg/dl)**			
Women	95.00 (89.00-101.00)	93.00 (88.00-100.00)	0.1607
Men	96.00 (92.00-104.00)	96.00 (91.00-102.00)	0.2750
**Uric acid (mg/dl)**			
Women	4.76 (4.22-5.35)	4.78 (4.06-5.34)	0.8241
Men	6.53 (5.60-7.48)	6.39 (5.66-7.27)	0.4740
**Serum creatinine (mg/dl)**			
Women	.70 (.62-.78)	.72 (.65-.78)	0.1661
Men	.98 (.88-1.06)	.95 (.87-1.02)	0.2424
**Urine creatinine (mg/24 h)**			
Women	1025.83 (866.04-1330.60)	1036.36 (859.98-1218.10)	0.1977
Men	1711.77 (1467.72-1947.12)	1592.64 (1319.61-1872.64)	0.0587
**Serum sodium (mmol/l)**			
Women	137.00 (136.00-139.00)	137.00 (136.00-139.00)	0.7914
Men	138.00 (137.00-140.00)	138.00 (137.00-139.00)	0.3052
**Urine sodium (mg/24 h)**			
Women	116.53 (81.82-165.10)	115.23 (87.86-149.83)	0.5057
Men	148.13 (114.42- 190.55)	149.66 (108.70-192.85)	0.5822
**Seric iron (mu g/dl)** [c]			
Women	89.95 (63.30-116.80)	93.6 (68.2-121.4)	0.2380
Men	110.45 (87.60-142.60)	118.45 (97.9-147.5)	0.1186

[a] *P values* were obtained using Pearson's chi-squared test or Wilcoxon test accordingly.
[b] The values are expressed in n (%) and [c] med (IQR 25-75).

Table 6. Sleep characteristics (1) among to hypertension and sex and age matched normotension participant

Characteristics	Hypertension (150)	Normotension (600)	*P value* [a]
**Sleep not calm (Sometimes)**			
Women	30 (20.00)	127 (21.20)	0.8400
Men	17 (11.33)	80 (13.36)	0.6052
**Sleep not calm (Hardly ever)**			
Women	15 (10.00)	87 (14.52)	0.1919
Men	9 (6.00)	54 (9.02)	0.3076
**Sleep not calm (Almost always)**			
Women	10 (6.67)	39 (6.51)	0.9999
Men	3 (2.00)	21 (3.51)	0.5001
**Sleep not calm (Many times)**			
Women	12 (8.00)	22 (3.67)	0.0392
Men	4 (2.67)	17 (2.84)	0.9999
**Sleep not calm (Never)**			
Women	19 (12.67)	68 (11.35)	0.7538
Men	16 (10.67)	50 (8.45)	0.4586
**Sleep not calm (Always)**			
Women	6 (4.00)	25 (4.17)	0.9999
Men	9 (6.00)	9 (1.50)	0.0035
**SBUAH (Sometimes)**			
Women	24 (16.00)	69 (11.52)	0.1747
Men	16 (10.67)	26 (4.34)	0.0048
**SBUAH (Hardly ever)**			
Women	17 (11.33)	46 (7.68)	0.1993
Men	7 (4.67)	33 (5.51)	0.8390
**SBUAH (Almost always)**			
Women	8 (5.33)	4 (0.67)	0.0002
Men	0 (0)	1 (0.17)	0.9999
**SBUAH (Many times)**			
Women	3 (2.00)	7 (1.17)	0.6907
Men	2 (1.33)	4 (0.67)	0.7585
**SBUAH (Never)**			
Women	40 (26.67)	239 (39.90)	0.0039
Men	33 (22.00)	167 (27.88)	0.1797
**SBUAH (Always)**			
Women	0 (0)	3 (0.50)	0.9999
Men	0 (0)	0 (0)	NA
**Sleepy during the day (Sometimes)**			
Women	42 (28.00)	153 (25.54)	0.6029
Men	34 (22.67)	117 (19.53)	0.4525
**Sleepy during the day (Hardly ever)**			
Women	10 (6.67)	59 (9.85)	0.2973
Men	8 (5.33)	51 (8.51)	0.2632
**Sleepy during the day (Almost always)**			
Women	11 (7.33)	38 (6.34)	0.7960
Men	3 (2.00)	12 (2.00)	0.9999
**Sleepy during the day (Many times)**			
Women	16 (10.67)	53 (8.85)	0.5913
Men	5 (3.33)	19 (3.17)	0.9999

[a] *P values* were obtained using Pearson's chi-squared test. All the values are expressed in n (%). *SBUA*, Shortness of breath upon awakening and headache

Table 7. Sleep characteristics (2) among to hypertension and sex and age matched normotension participant

Characteristics	Hypertension (150)	Normotension (600)	*P value* [a]
**Sleepy during the day (Never)**			
Women	11 (7.33)	46 (7.68)	0.9999
Men	7 (4.67)	30 (5.01)	0.9999
**Sleepy during the day (Always)**			
Women	2 (1.33)	19 (3.17)	0.3469
Men	1(0.67)	2 (0.33)	0.9999
**Trouble sleeping (Sometimes)**			
Women	24 (16.00)	103 (17.20)	0.8266
Men	15 (10.00)	56 (9.35)	0.9255
**Trouble sleeping (Hardly ever)**			
Women	18 (12.00)	94 (15.69)	0.3178
Men	16 (10.67)	61 (10.18)	0.9760
**Trouble sleeping (Almost always)**			
Women	7 (4.67)	24 (4.01)	0.8906
Men	2 (1.33)	12 (2.00)	0.8396
**Trouble sleeping (Many times)**			
Women	11 (7.33)	16 (2.67)	0.0125
Men	3 (2.00)	10 (1.67)	0.9999
**Trouble sleeping (Never)**			
Women	27 (18.00)	108 (18.03)	0.9999
Men	21 (14.00)	85 (14.19)	0.9999
**Trouble sleeping (Always)**			
Women	5 (3.33)	23 (3.84)	0.9616
Men	1 (0.67)	7 (1.17)	0.9292
**Difficulty going back to sleep (Sometimes)**			
Women	33 (22.00)	111 (18.53)	0.3911
Men	23 (15.33)	68 (11.35)	0.2293
**Difficulty going back to sleep (Hardly ever)**			
Women	20 (13.33)	93 (15.53)	0.5920
Men	10 (6.67)	67 (11.19)	0.1406
**Difficulty going back to sleep (Almost always)**			
Women	5 (3.33)	26 (4.34)	0.7482
Men	2 (1.33)	9 (1.50)	0.9999
**Difficulty going back to sleep (Many times)**			
Women	11 (7.33)	30 (5.01)	0.3557
Men	1 (0.67)	7 (1.17)	0.9292
**Difficulty going back to sleep (Never)**			
Women	22 (14.67)	94 (15.69)	0.8597
Men	22 (14.67)	75 (12.52)	0.5678
**Difficulty going back to sleep (Always)**			
Women	1 (0.67)	14 (2.34)	0.3280
Men	0 (0)	5 (0.83)	0.5749
**Stay awake during the day (Sometimes)**			
Women	12 (8.00)	71 (11.85)	0.2329
Men	14 (9.33)	49 (8.18)	0.7671
**Stay awake during the day (Hardly ever)**			
Women	30 (20.00)	113 (18.86)	0.8343
Men	18 (12.00)	76 (12.69)	0.9341

[a] *P values* were obtained using Pearson's chi-squared test. All the values are expressed in n (%).

Table 8. Sleep characteristics (3) among to hypertension and sex and age matched normotension participant

**Characteristics**	**Hypertension (150)**	**Normotension (600)**	***P value*** [a]
**Stay awake during the day (Almost always)**			
Women	3 (2.00)	6 (1.00)	0.5573
Men	1 (0.67)	3 (0.50)	0.9999
**Stay awake during the day (Many times)**			
Women	4 (2.67)	10 (1.67)	0.6368
Men	2 (1.33)	5 (0.83)	0.9244
**Stay awake during the day (Never)**			
Women	43 (28.67)	165 (27.55)	0.8544
Men	23 (15.33)	98 (16.36)	0.8621
**Stay awake during the day (Always)**			
Women	0 (0)	3 (0.50)	0.9999
Men	0 (0)	0 (0)	NA
**Snores while sleeping (Sometimes)**			
Women	34 (22.67)	144 (24.04)	0.8134
Men	27 (18.00)	87 (14.52)	0.3468
**Snores while sleeping (Hardly ever)**			
Women	8 (5.33)	49 (8.18)	0.3178
Men	4 (2.67)	22 (3.67)	0.7269
**Snores while sleeping (Almost always)**			
Women	16 (10.67)	36 (6.01)	0.0668
Men	10 (6.67)	33 (5.51)	0.7238
**Snores while sleeping (Many times)**			
Women	3 (2.00)	19 (3.17)	0.6263
Men	6 (4.00)	23 (3.84)	0.9999
**Snores while sleeping (Never)**			
Women	21 (14.00)	86 (14.36)	0.9999
Men	7 (4.67)	27 (4.51)	0.9999
**Snores while sleeping (Always)**			
Women	10 (6.67)	34 (5.68)	0.7857
Men	4 (2.67)	39 (6.51)	0.1074
**Naps during the day (Sometimes)**			
Women	31 (20.67)	126 (21.04)	0.9999
Men	26 (17.33)	97 (16.19)	0.8244
**Naps during the day (Hardly ever)**			
Women	20 (13.33)	68 (11.35)	0.5899
Men	6 (4.00)	43 (7.18)	0.2228
**Naps during the day (Almost always)**			
Women	6 (4.00)	17 (2.84)	0.6337
Men	7 (4.67)	12 (2.00)	0.1168
**Naps during the day (Many times)**			
Women	5 (3.33)	21 (3.51)	0.9999
Men	2 (1.33)	18 (3.01)	0.3954
**Naps during the day (Never)**			
Women	30 (20.00)	131 (21.87)	0.7055
Men	15 (10.00)	53 (8.85)	0.7748
**Naps during the day (Always)**			
Women	0 (0)	5 (0.83)	0.5749
Men	2 (1.33)	8 (1.34)	0.9999

[a] *P values* were obtained using Pearson's chi-squared test. All the values are expressed in n (%).

Table 9. Sleep characteristics (4) among to hypertension and sex and age matched normotension participant

**Characteristics**	**Hypertension (150)**	**Normotension (600)**	***P value*** [a]
**Time it took to fall asleep (min)**			
Women			
0-15	51 (34)	221 (36.83)	0.5819
16-30	14 (9.33)	75 (12.50)	0.3516
31-45	9 (6)	14 (2.33)	0.0389
46-60	4 (2.67)	17 (2.83)	0.9999
over 60	14 (9.33)	41 (6.83)	0.3813
Men			
0-15	31 (20.67)	159 (26.50)	0.1725
16-30	13 (8.67)	36 (6.00)	0.3186
31-45	6 (4.00)	8 (1.33)	0.0685
46-60	3 (2.00)	9 (1.50)	0.9999
over 60	5 (3.3)	19 (3.17)	0.9999
**Get enough sleep (Sometimes)**			
Women	22 (14.67)	98 (16.36)	0.7088
Men	16 (10.67)	56 (9.33)	0.7332
**Get enough sleep (Hardly ever)**			
Women	18 (12.00)	64 (10.66)	0.7476
Men	6 (4.00)	37 (6.16)	0.4096
**Get enough sleep (Almost always)**			
Women	25 (16.67)	89 (14.83)	0.6656
Men	17 (11.33)	64 (10.66)	0.9297
**Get enough sleep (Many times)**			
Women	5 (3.33)	24 (4.00)	0.8870
Men	4 (2.67)	20 (3.34)	0.8763
**Get enough sleep (Never)**			
Women	10 (6.67)	54 (9.00)	0.4523
Men	4 (2.67)	20 (3.34)	0.8763
**Get enough sleep (Always)**			
Women	12 (8.00)	39 (6.50)	0.6374
Men	11 (7.33)	34 (5.67)	0.5642
**Optimal sleep (Good 1)**			
Women	36 (24.00)	152 (25.33)	0.8168
Men	31 (20.67)	101 (16.83)	0.3257
**Optimal sleep (Bad 0)**			
Women	56 (37.33)	216 (36.00)	0.8346
Men	27 (18.00)	131 (21.83)	0.3587
**Apnea (No)**			
Women	92 (61.33)	363 (60.60)	0.9256
Men	58 (38.67)	227 (37.90)	0.9251
**Apnea (Si)**			
Women	0 (0)	5 (0.83)	0.5749
Men	0 (0)	4 (0.67)	0.7069

[a] *P values* were obtained using Pearson's chi-squared test. All the values are expressed in n (%).

Table 10. Distribution of complete blood count (1) among to hypertension and sex and age matched normotension participant

Characteristics	Hypertension (150)	Normotension (600)	*P value* [a]
**Erythrocytes**			
Women	4.72 (4.52-4.96)	4.71 (4.50-4.97)	0.7799
Men	5.40 (5.15-5.74)	5.34 (5.14-5.59)	0.1306
**Hemoglobin (g/dL)**			
Women	14.20 (13.60-14.65)	14.20 (13.50-14.80)	0.9384
Men	16.55 (16.00-17.10)	16.40 (15.80-16.90)	0.1282
**Hematocrit (%)**			
Women	42.20 (40.35-43.80)	42.20 (40.40-44.10)	0.9557
Men	48.80 (46.90-50.70)	48.45 (46.70-49.90)	0.1614
**MCV (fl)**			
Women	89.90 (87.00-91.80)	89.70 (86.80-92.20)	0.8727
Men	89.80 (87.40-92.40)	90.45 (88.25-92.80)	0.3905
**MCH (Pg)**			
Women	30.30 (28.95-31.05)	30.10 (28.90-31.20)	0.9073
Men	30.50 (29.60-31.40)	30.50 (29.85-31.40)	0.5667
**MCHC (g/dL)**			
Women	33.50 (33.10-33.85)	33.50 (33.00-34.00)	0.9233
Men	33.80 (33.40-34.30)	33.80 (33.50-34.10)	0.9155
**Leukocytes**			
Women	6.60 (5.45-8.10)	6.20 (5.30-7.40)	0.0354
Men	6.60 (5.60-7.30)	6.20 (5.40-7.20)	0.2545
**Neutrophils (%)**			
Women	58.80 (53.90-64.10)	58.30 (52.90-63.30)	0.0430
Men	56.75 (52.60-63.10)	56.95 (50.85-61.60)	0.2010
**Neutrophils**			
Women	3.95 (2.90-5.10)	3.60 (2.90-4.40)	0.5619
Men	3.70 (3.00-4.30)	3.50 (2.80-4.20)	0.2758
**Lymphocytes (%)**			
Women	31.80 (26.70-35.40)	31.40 (26.70-36.10)	0.0756
Men	32.95 (26.90-36.50)	32.95 (27.75-37.10)	0.8498

[a] *P values* were obtained using Wilcoxon test. All the values are expressed in med (IQR 25-75). *MCV*, mean corpuscular volume; *MCH*, mean corpuscular hemoglobin; *MCHC* mean corpuscular hemoglobin concentration.

Table 11. Distribution of complete blood count (2) among to hypertension and sex and age matched normotension participant

Characteristics	Hypertension (150)	Normotension (600)	*P value* [a]
**Lymphocytes**			
Women	2.10 (1.65-2.50)	2.00 (1.60-2.30)	0.7370
Men	2.00 (1.70-2.40)	2.00 (1.60-2.40)	0.5799
**Monocytes (%)**			
Women	6.40 (5.40-7.55)	6.60 (5.70-7.70)	0.0784
Men	7.00 (6.00-8.50)	7.20 (6.20-8.40)	0.4177
**Monocytes**			
Women	.50 (.30-.50)	.40 (.30-.50)	0.3169
Men	.50 (.40-.60)	.50 (.40-.60)	0.7422
**Eosinophils (%)**			
Women	2.20 (1.40-3.50)	2.30 (1.50-3.50)	0.4418
Men	2.45 (1.70-3.10)	2.70 (1.80-4.10)	0.1103
**Eosinophils**			
Women	.15 (.10-.20)	.10 (.10-.20)	0.6713
Men	.10 (.10-.20)	.20 (.10-.30)	0.0787
**Basophils (%)**			
Women	0 (0)	0 (0)	0.1254
Men	0 (0)	0 (0)	0.6193
**Basophils**			
Women	.50 (.40-.60)	.50 (.40-.60)	0.6931
Men	.50 (.40-.60)	.50 (.40-.60)	0.4310
**Platelets**			
Women	275.00 (232.00-333.00)	267.00 (231.00-307.00)	0.2672
Men	320.50 (203.00-263.00)	227.00 (198.00-268.00)	0.6804
**MPV (fL)**			
Women	8.55 (8.00-9.15)	8.40 (7.80-9.10)	0.1972
Men	8.60 (7.90-9.3)	8.60 (8.00-9.20)	0.6976
**RDW (%)**			
Women	13.65 (13.25-14.40)	13.60 (13.20-14.40)	0.5108
Men	13.50 (13.20-13.80)	13.40 (13.10-13.80)	0.8612

[a] *P values* were obtained using Wilcoxon test. All the values are expressed in med (IQR 25-75). *MPV* Mean platelet volume; *RDW*, Red blood cell distribution width.

Table 12 Nutrient Composition

Variable	Nutrient Composition	Unit
CALOR	energy	kilocalories
PROTEI	total proteins	grams
APROT	animal origin proteins	grams
CARBO	carbohydrates	grams
SUCR	sucrose	grams
FRUCT	fructose	grams
LACT	lactose	grams
ST	starch	grams
MALT	maltose	grams
GLU	glucose	grams
CRUDE	crude fiber	grams
DTFIB	dietary fiber Southgate	grams
AOFID	AOAC fiber	grams
ENGL	Englyst type fiber	grams
SOLFB	soluble dietary fiber	grams
PECT	pectin	grams
INSFB	insoluble dietary fiber	grams
CELLU	cellulose	grams
LIGN	lignin	grams
CALC	calcium	milligrams
IRON	total iron	milligrams
HEMCL	heme iron	milligrams
HEME	hemicellulose	milligrams
MAGN	magnesium	milligrams
PH	phosphorus	milligrams
K	potassium	milligrams
SODIUM	sodium	milligrams
ZN	zinc	milligrams
CU	copper	milligrams
MN	manganese	milligrams
IODINE	iodine	micrograms
SE	selenium	micrograms
VITC	vitamin C	milligrams
B1	thiamine	milligrams
B2	riboflavin	milligrams
NIACIN	niacin	milligrams
PANTO	pantothenic acid	milligrams
B6	vitamin B6	milligrams
GLYB6	glucosylated vitamin B6	milligrams
FOLATE	folates	micrograms
B12	vitamin B12	micrograms

Table 12 continued from previous page

Variable	Nutrient Composition	Unit
VITK	vitamin K	micrograms
RETINOL	retinol	Units
CAROT	carotenoids	Units
ACAR	alpha-carotenes	micrograms
BCAR	beta-carotenes	micrograms
BCRYP	beta-cryptoxanthins	micrograms
LYCO	lycopene	micrograms
LUT	lutein + xanthins	micrograms
VITD	vitamin D	Units
VITE	vitamin E	micrograms
VITEIU	vitamin E activity	Units
ATOCO	alpha-tocopherol	milligrams
BTOCO	beta-tocopherol	milligrams
GTOCO	gamma-tocopherol	milligrams
DTOCO	delta-tocopherol	milligrams
TTOCO	total tocopherols	milligrams
ATEQ	alpha-tocopherol equivalents	equivalents mg TE
CHOL	cholesterol	grams
ALCO	alcohol	grams
CAFF	caffeine	grams
NITRATE	nitrates	grams
AFAT	animal origin fat	grams
VFAT	vegetable origin fat	grams
TFATAV	total fat: animal + vegetable	grams
SATFAT	saturated fat	grams
MONFAT	monounsaturated fat	grams
POLY	polyunsaturated fat	grams
F40	butyric acid 4.0	grams
F60	fatty acid 6.0	grams
F80	caprylic acid 3.0	grams
F100	capric acid 10.0	grams
F120	fatty acid 12.0	grams
F12091	lauric acid	grams
F140	fatty acid 14.0	grams
F14091	myristic acid	grams
F160	fatty acid 16.0	grams
F16091	palmitic acid	grams
F180	stearic acid 18.0	grams
F18091	stearic acid	grams
F161	palmitoleic acid 16.1	grams
F16T191	fatty acid 16.1	grams
F181	oleic acid	grams

Table 12 continued from previous page

Variable	Nutrient Composition	Unit
CIS18191	cis fatty acid 18.1	grams
TR18191	trans fatty acid 18.1	grams
F201	fatty acid 20.1	grams
F221	F221	grams
F182	linolenic acid	grams
CIS18291	cis fatty acid 18.2	grams
TR18291	trans fatty acid 18.2	grams
CT18291	cis fatty acid 18.2	grams
F183	linolenic acid 18.3	grams
F18391	linolenic acid	grams
F184	F184	grams
F204	arachidonic acid 20.4	grams
F20491	arachadonic acid	grams
F205	fatty acid 20.5	grams
F20591	EPA	grams
F225	fatty acid 22.5	grams
F226	DHA fatty acid 22.6	grams
F22691	DHA fatty acid	grams
TFATSMP	Total fats: mono + poly + satur	grams
